# Design and Theoretical Analysis of a Dynamically Tunable Terahertz Broadband Absorber Based on a Graphene/MoS_2_ Vertical Heterojunction and VO_2_ Phase Transition

**DOI:** 10.3390/ma19214705

**Published:** 2026-11-05

**Authors:** Xiaoyue Lu, Xianbin Zhang, Shihan Zhao, Huiyu Liu

**Affiliations:** School of Optoelectronic Science and Intelligent Instruments, Xi’an University of Technology, Xi’an 710054, China; luxy457764033@outlook.com (X.L.); unicornzsh@163.com (S.Z.); 18191221090@163.com (H.L.)

**Keywords:** terahertz absorber, graphene/MoS_2_ heterojunction, VO_2_ phase transition, broadband absorption, coupled mode theory, dynamic tuning

## Abstract

**Highlights:**

- A graphene/MoS_2_/VO_2_ absorber achieves 91.7% absorption from 3.5 to 6.5 THz.
- VO_2_ phase transition provides coarse switching between broadband and reflection.
- Graphene Fermi level enables continuous fine-tuning of impedance matching.
- MoS_2_ enhances local field confinement and low-loss Ohmic dissipation.
- CMT and unit removal analysis reveal three-mode synergy and nonlinear coupling.

**Abstract:**

This paper proposes a tunable terahertz broadband absorber based on a graphene/MoS_2_ vertical heterojunction and a VO_2_ phase-change film. VO_2_ reconfigures the longitudinal Fabry–Perot cavity via its insulator-metal transition, switching between broadband absorption and weak reflection. graphene adjusts its Drude conductivity and kinetic inductance through its Fermi level, compensating for band-edge impedance mismatch and broadening the absorption band. MoS_2_ enhances local field confinement and Ohmic dissipation via intraband free-carrier response and interface charge transfer. The three materials form a synergistic mechanism of phase-change coarse tuning, electrical fine-tuning, and loss enhancement. Results show that in the VO_2_ insulating state, absorption exceeds 91.7% from 3.5 to 6.5 THz, with a relative bandwidth of 60.8%. Coupled mode theory decomposes the broadband absorption into three under-coupled modes, corresponding to low-frequency anchoring, mid-frequency bridging, and high-frequency enhancement. Unit peeling analysis shows that the eight asymmetric units achieve nonlinear synergy through near-field coupling and current topology reconstruction, rather than simple superposition. The device also exhibits less than 2% degradation under ±20% processing deviation. It maintains over 91.7% absorption within 0–60° incidence under TE polarization, demonstrating good process robustness and angle stability.

## 1. Introduction

Terahertz (THz) waves typically refer to electromagnetic waves with frequencies ranging from 0.1 to 10 THz, lying between microwaves and infrared. They possess characteristics such as penetrability, coherence, and fingerprint spectra. They have significant application value in broadband communication, biochemical sensing, nondestructive testing, and security protection. However, the THz frequency band lacks high-performance functional devices, which hinders its practical application. Metamaterial absorbers are an important component of THz systems, and their performance affects the detection sensitivity and control controllability of the system [1].

Traditional metal-based metamaterial absorbers usually adopt a sandwich structure of metal resonator layer/dielectric spacer layer/metal ground plane, achieving high absorption through impedance matching [1]. However, these devices have three limitations: (1) The absorption bandwidth is limited. The absorption peaks produced by a single resonant mechanism are narrow and sharp, and the relative bandwidth is usually insufficient. (2) They lack dynamic tuning capability. The electromagnetic response is determined by geometric parameters, and it is difficult to change after fabrication. (3) It is difficult to balance broadband absorption, dynamic tuning, and angle stability.

To expand the absorption bandwidth and introduce active control, researchers adopted schemes such as multi-layer stacking [2,3], coplanar multi-resonant units [4,5], and fractal structures [6]. However, the fabrication process of multi-layer stacking structures is complex, and strict alignment accuracy is required between layers. In addition, symmetrical structures are restricted by selection rules, and higher-order dark modes cannot be directly excited by external plane waves. This results in a limited number of available resonant modes and a Q-value–bandwidth contradiction [7,8]. At the material selection level, introducing new functional materials is the core approach to achieving active control. Graphene has the characteristic of an adjustable Fermi level ($E_{F}$), along with a zero bandgap, which can provide continuous electrical control [9,10]. Molybdenum disulfide has a direct bandgap of approximately 1.8 eV, with low intrinsic loss in the terahertz frequency range, and can be used as a low-loss optical response enhancement layer [11]. Vanadium dioxide exhibits a conductivity variation of about three orders of magnitude during the insulator–metal phase transition and can be used for macroscopic mode switching [12,13,14].

Dynamic terahertz modulation can also be achieved through various active mechanisms other than two-dimensional materials and phase-change oxides. For instance, an ultrathin surface based on high-temperature superconductors (such as YBCO) can achieve ultrafast switching by destroying Cooper pairs through thermal or optical pumping, but it requires cryogenic cooling below approximately 90 K, which significantly limits its practical integration applications [15]. The tuning technology based on liquid crystals can achieve continuous refractive index modulation through the induced molecular rearrangement by an external electric field, but it requires relatively thick liquid crystal units [16]. Conductive polymers (such as PEDOT: 0PSS) have excellent flexibility and low-cost electrochemical tuning potential, but their electrochemical processes of charge insertion and removal are slow, with slow switching speeds (often on the order of milliseconds to seconds, depending on device geometry and electrolyte) [17]. Chalcogenide phase-change materials, such as Ge_2_Sb_2_Te_5_ (GST) and Ge_2_Sb_2_Se_4_Te_1_ (GSST), have a wide range and non-volatile optical contrast and have been widely used in reconfigurable photonic/terahertz devices [18,19], but their switching relies on external optical or electrical pulses to drive the transition between amorphous and crystalline states. The crystallization process is relatively long (microseconds to milliseconds) and energy-intensive, which may lead to a decrease in cycle stability. In contrast, graphene provides a continuous and electrically controllable Fermi level, with carrier response times lower than picoseconds. Two-dimensional molybdenum disulfide (MoS_2_) provides a low-loss, direct bandgap platform for interlayer charge transfer and field confinement. Vanadium dioxide (VO_2_) exhibits rapid (picosecond- to nanosecond-scale), reversible, and volatile insulator-metal transitions near the critical temperature, thereby achieving a switch based on resistance/optical heating. Therefore, this complementary combination of continuous electrically controllable regulation, low-loss field enhancement, and stable reversible phase transitions effectively resolves the contradiction between modulation depth, response speed, and system power consumption, providing a feasible path for realizing a broadband terahertz absorber that is reconfigurable both electrically and thermally, which is precisely the goal of this design.

In recent years, the integration of dual materials has become an important approach for expanding the tuning capabilities of terahertz absorbers. The graphene/VO_2_ system, through electrical regulation and phase change switching, has been reported to have achieved broadband absorption and dynamic tuning [20,21]. Chen W. et al. (2022) utilized the synergistic regulation of graphene/VO_2_ to achieve switchable broadband (over 95%, 2.85–10 THz) and narrowband absorption [22]. Wen Hao et al. (2025) proposed a five-layer graphene/VO_2_ structure that achieved ultra-wideband absorption in the range of 2.05–14.03 THz [21]. The graphene/MoS_2_ heterojunction can enhance the low-loss optical response through interlayer charge transfer and $E_{F}$ regulation [23,24]. He Jiayi et al. (2026) proposed a graphene/MoS_2_ complementary metamaterial absorber that achieved broadband absorption in the range of 2.1–3.9 THz and could switch between narrowband sensing and ultra-wideband absorption modes through two-dimensional electrical tuning [25]. However, the tunability of existing dual-material systems is still limited: the graphene/VO_2_ system lacks a mechanism for enhancing low-loss optical response, the graphene/MoS_2_ system lacks a significant switching modulation capability, and the MoS_2_/VO_2_ system has received relatively less research in the terahertz frequency range [26]. At present, there are still relatively few studies that have successfully integrated graphene, MoS_2_ and VO_2_ simultaneously into a single supermaterial structure and achieved coordinated regulation of electrical modulation, low-loss enhancement and phase-change switching [27].

In response to the above issues, this paper proposes a terahertz broadband tunable absorber composed of a graphene/MoS_2_ vertical heterojunction and a VO_2_ phase-change film. In view of the inherent trade-off between the macroscopic switching ratio and the continuous fine-tuning capability in traditional dual-material systems, this design, for the first time, constructs a three-material co-regulation structure of phase-change coarse-tuning—electric control fine-tuning—polarization excitation enhancement. The broadband absorption performance of the device in the VO_2_ insulating state was realized through FDTD full-wave simulation, and the broadband absorption was decomposed into three under-coupled resonant modes using the coupled-mode theory. Moreover, through structural analysis and material stripping analysis, the cooperative mechanism of the asymmetric unit in near-field coupling and current topological reconstruction was revealed. At the same time, the control capabilities of VO_2_ phase change, graphene $E_{F}$ and MoS_2_ carrier concentration on the absorption spectrum were systematically analyzed, providing a new design idea for high-performance terahertz dynamic metamaterial devices with multiple external stimulus responses.

## 2. Structural Design and Theoretical Model

### 2.1. Structure Design

**Figure 1:**
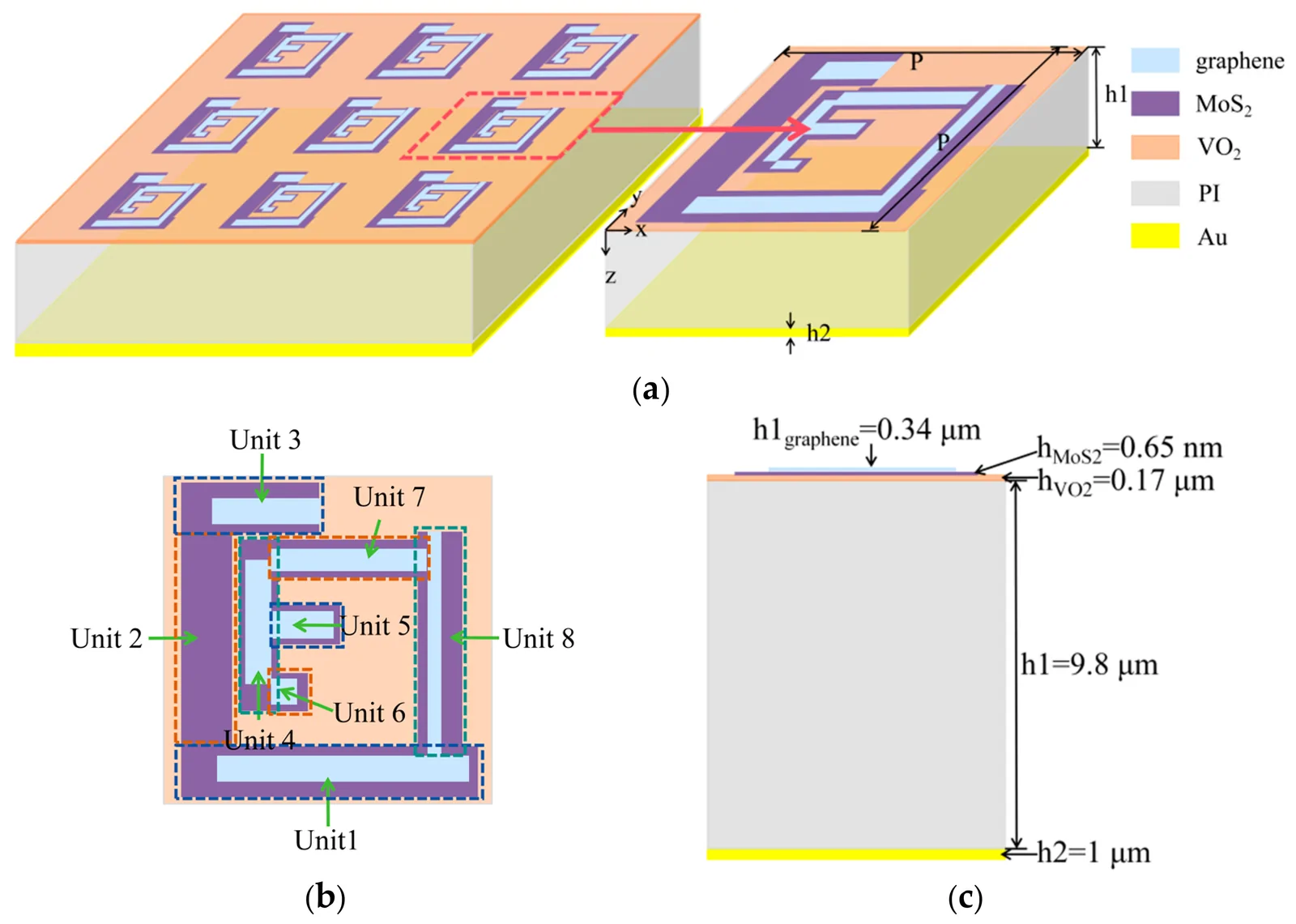
Schematic of a graphene/MoS_2_ vertical heterostructure—VO_2_ phase-change terahertz absorber: (**a**) Three-dimensional perspective view. (**b**) Top view. (**c**) Cross-sectional (x–z) stack diagram of the unit cell, showing the layer sequence and thicknesses of the Au reflector, PI dielectric layer, VO_2_ film, and graphene/MoS_2_ heterojunction pattern.

The tunable terahertz broadband absorber unit cell structure designed in this section is shown in Figure 1, with a period (P) of 35 μm. The entire device adopts an asymmetric structural design, consisting of a metal reflective substrate (gold), a polyimide(PI) dielectric layer, a continuous VO_2_ phase-change film layer, and a top graphene/MoS_2_ vertical heterostructure pattern layer from bottom to top.

In terms of geometric design, the two-dimensional material heterostructure at the top adopts an asymmetric nested pattern, and a continuous VO_2_ film layer with a thickness of 0.17 μm is added between the heterostructure pattern layer and the PI dielectric layer. This layout does not require patterning and etching of VO_2_, while enabling VO_2_ to act as a continuous impedance control layer and interact with the local field of the top heterostructure.

In this paper, the electromagnetic response of the structure was numerically simulated using the Lumerical FDTD Solutions software based on the finite-difference time-domain method.

The geometric parameters and spatial distribution of each structural layer are as follows:

The top layer is a graphene/MoS_2_ heterojunction pattern layer, consisting of 8 units. The geometric parameters of each unit are shown in Table 1:

The geometric structure of eight asymmetric graphene/molybdenum disulfide units was obtained through a sequential parameter scanning design strategy, rather than an arbitrary layout. The length L of each unit in the planar direction is approximately in accordance with the first-order local dipole resonance condition, so as to simultaneously cover the low, medium, and high frequency bands. Therefore, the final design consists of eight units.

The dielectric layer is made of PI, with a thickness of h1 = 9.8 μm. The complex refractive index of PI also evolves with frequency and is incorporated into the simulation model. Within the frequency range of 1.0–7.0 THz, the real part n of the refractive index $n\left(\omega \right)=n+ik$ of PI fluctuates slightly between 1.82 and 1.73, while the imaginary part k (absorption coefficient) remains within the range of 0.015 to 0.045. The PI dielectric layer acts as the spacer between the top graphene/MoS_2_ heterojunction pattern and the bottom metal reflective layer. By adjusting the near-field vertical coupling strength, the impedance matching conditions of each resonant mode are optimized. This also provides vertical dimension support for multi-mode resonances. The bottom layer is a gold reflective layer with a thickness of h2 = 1 μm, and a conductivity $\sigma =4\times 10^{7}$. Its thickness is much larger than the skin depth (approximately 100 nm), and the transmission *T(ω)* ≈ 0.

In terms of boundary conditions, the x and y directions are set as periodic boundaries, while the z direction is set as a perfectly matched layer. The grid size is set to 0.2×0.2×0.1 μm, and the total simulation duration is 3000 fs. For the graphene/MoS_2_ atomic-level material layer, a locally refined grid with Δz = 0.0001 μm is adopted.

The heterogeneous superstructure proposed in this paper is experimentally feasible under the current micro–nano processing capabilities. The manufacturing technology route from the bottom layer to the top layer is as follows: (1) Deposit a 1 μm thick gold grounding plane layer on the substrate by electron beam evaporation or sputtering. (2) Spin-coat and solidify a 9.8 μm thick polyimide dielectric layer. (3) Deposit a continuous, non-patterned 0.17 μm thick VO_2_ film on the polyimide surface by magnetron sputtering or pulsed laser deposition, followed by annealing. (4) Transfer the single-layer graphene and single-layer MoS_2_ grown by chemical vapor deposition to the VO_2_ surface by wet or dry transfer methods. (5) Use electron beam lithography to define an eight-unit asymmetric pattern, then perform O_2_ plasma etching on the graphene and reactive ion etching on the MoS_2_. (6) Deposit ion liquid gate electrodes and contact pads in the graphene/MoS_2_ system to achieve electrical bias. These steps are compatible with the standard cleanroom process, and the continuous non-patterned VO_2_ film eliminates the need for additional patterning and alignment steps.

### 2.2. Theoretical Model

This paper employs the Drude-type dispersion model to describe the electromagnetic responses of graphene, MoS_2_, and VO_2_ in the terahertz frequency range. The material parameters are calibrated based on experimental data at room temperature (T = 300 K). Each of the three materials provides a phase change switch, continuous electrical regulation, and low-loss optical enhancement.

(1) graphene

In the terahertz frequency range ($\hbar \omega \ll E_{F}$, $E_{F}\gg k_{B}T$), the contribution of band-to-band transitions can be neglected, and the surface conductivity of graphene can be approximated in the Drude form [28]:

(1) $$\sigma_{g}\left(\omega \right)=\frac{ie^{2}E_{F}}{\pi \hbar^{2}\left(\omega +i/\tau \right)}$$

In the equation, ω represents angular frequency; $\mu_{c}$ is the chemical potential (Fermi energy level) of graphene; τ is the relaxation time.

The $E_{F}$ of graphene can be continuously adjusted by electrostatic gate control. In the top-gate structure, the relationship between the gate voltage $V_{g}$ and the carrier concentration n is:

(2) $$n=\frac{C_{ox}\left(V_{g}-V_{Dirac}\right)}{e}$$

In the equation, $C_{ox}=\varepsilon_{0}\varepsilon_{r}/t_{ox}$ represents the gate oxide capacitance per unit area; $V_{Dirac}$ is the Dirac point voltage (corresponding to $E_{F}=0$).

Substituting this equation into $E_{F}=\hbar v_{F}\sqrt{\pi n}$, we obtain:

(3) $$E_{F}=\hbar v_{F}\sqrt{\frac{\pi C_{ox}\left(V_{g}-V_{Dirac}\right)}{e}}$$

The ionic liquid gate utilizes the double-layer effect to provide an electric capacitance density of approximately 10 μF/cm^2^, and can be adjusted within 3 V to a range of 0–1.0 eV.

(2) MoS_2_

The complex electrical conductivity of single-layer MoS_2_ in the terahertz frequency range conforms to the Drude-like response characteristics. Due to the extremely low energy of terahertz photons compared to its band gap, interband transitions are suppressed, and the loss of free carriers within the band becomes the dominant mechanism [29]:

(4) $$\sigma =\frac{ne^{2}\tau }{m^{*}\left(1-i\omega \tau \right)}$$

In the equation, n represents the concentration of free charge carriers, defined as the ratio of surface density to equivalent thickness. e represents the electron charge (1.602 × 10 − 19 C); τ represents the relaxation time (scattering time, unit ps); $m^{*}$ represents the effective electron mass ($m^{*}=0.53m_{e}$), $m_{e}$ represents the rest mass of free electrons. In the simulation, $n=0.8\times 10^{14}-8\times 10^{14}$ is adopted. This range can be achieved through the ionic liquid gate.

(3) VO_2_

In the terahertz frequency range, the complex dielectric constant of VO_2_ is described by the Drude model [22]:

(5) $$\varepsilon \left(\omega \right)=\varepsilon_{\infty }-\frac{{\omega_{p}}^{2}}{\omega^{2}+i\gamma \omega }$$

In the equation, $\varepsilon_{\infty }=12$ is the high-frequency dielectric constant, which reflects the contribution of bound electrons to polarization and originates from interband transitions. γ=1/τ is the collision frequency (scattering rate), and τ is the relaxation time. This value is obtained by fitting the reflection spectrum of the metal state VO_2_; $\omega_{p}$ is the plasma frequency, and its functional relationship with the electrical conductivity σ is:

(6) $$\omega_{p}^{2}\left(\sigma \right)=\frac{\sigma }{\sigma_{0}}\omega_{p}^{2}\left(\sigma_{0}\right)$$

Model parameters: Insulating state $\sigma_{{\mathrm{VO}}_{2}}=30$ S/m (at room temperature 25 °C); Metallic state $\sigma_{{\mathrm{VO}}_{2}}=9\times 10^{4}$ S/m (at high temperature 75 °C).

## 3. Analysis and Discussion

### 3.1. Absorption Characteristics and Physical Mechanism

#### 3.1.1. Verification of Absorption Performance of the Complete Structure of Three Materials

**Figure 2:**
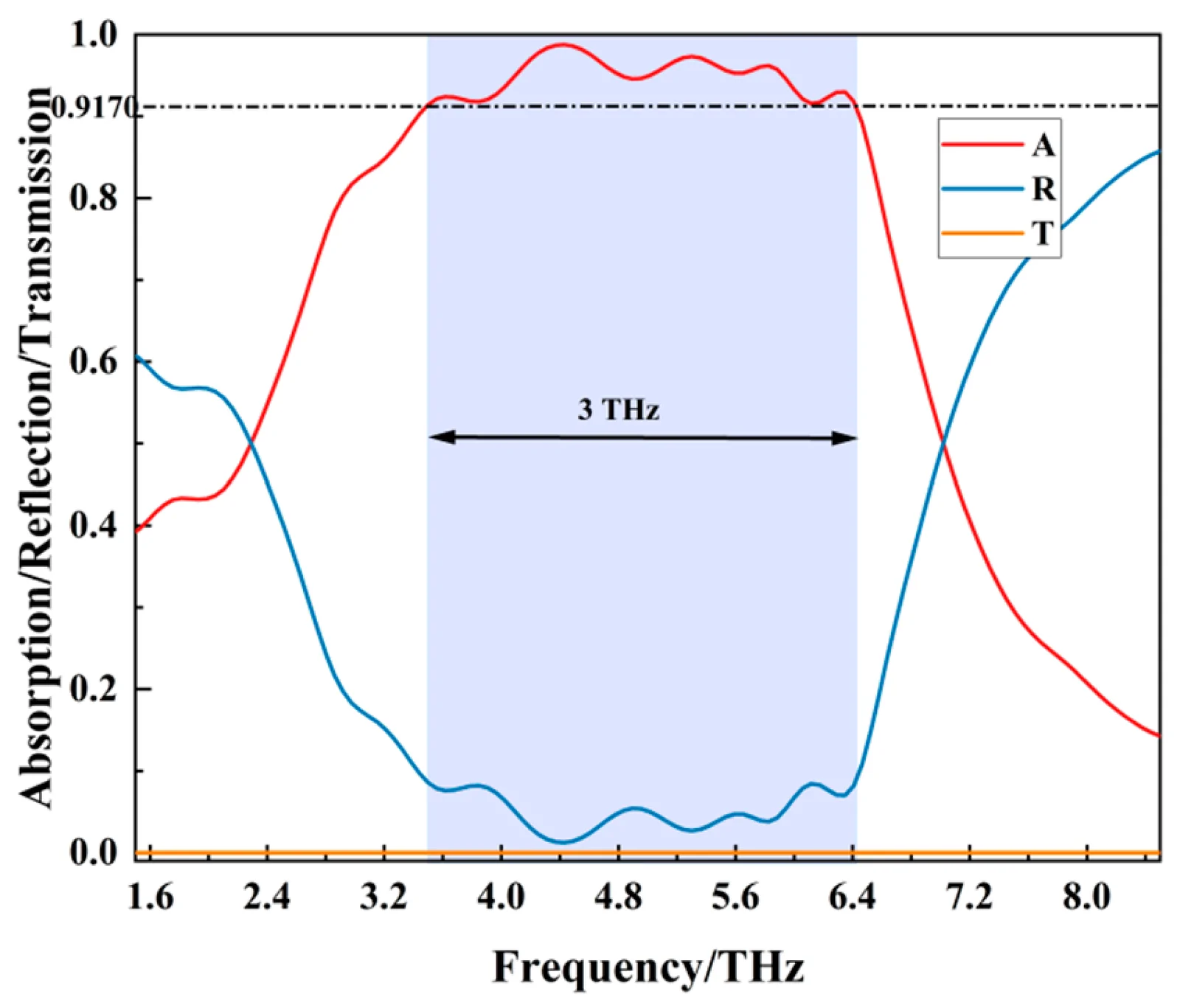
Absorption, reflection, and transmission spectra of the proposed absorber.

This section analyzes the absorption spectra of the graphene/MoS_2_ heterojunction and the VO_2_ composite structure in the terahertz band. Figure 2 presents the absorption, reflection, and transmission of this absorber in the range of 1.5–8.5 THz. The thickness of the underlying gold film is much greater than the skin depth, and the electromagnetic wave cannot penetrate, so *T(ω)* = 0. From *A(ω)* = 1 − *R(ω)* − *T(ω)*, it can be seen that the absorption is determined by the reflection. Within the range of 3.5–6.5 THz, the absorption is greater than 91.7%, forming a broadband high absorption region. This structure has the highest absorption at 4.43 THz, which is 98.75%; there is a secondary high absorption peak of 97.3% near 5.30 THz, indicating that multiple resonant modes are jointly involved in absorption. Its absolute absorption bandwidth is 2.98 THz, the center frequency is 4.93 THz, and the relative bandwidth is 60.8%. These results indicate that the device can achieve broadband absorption in the terahertz band.

#### 3.1.2. Impedance Matching Verification

**Figure 3:**
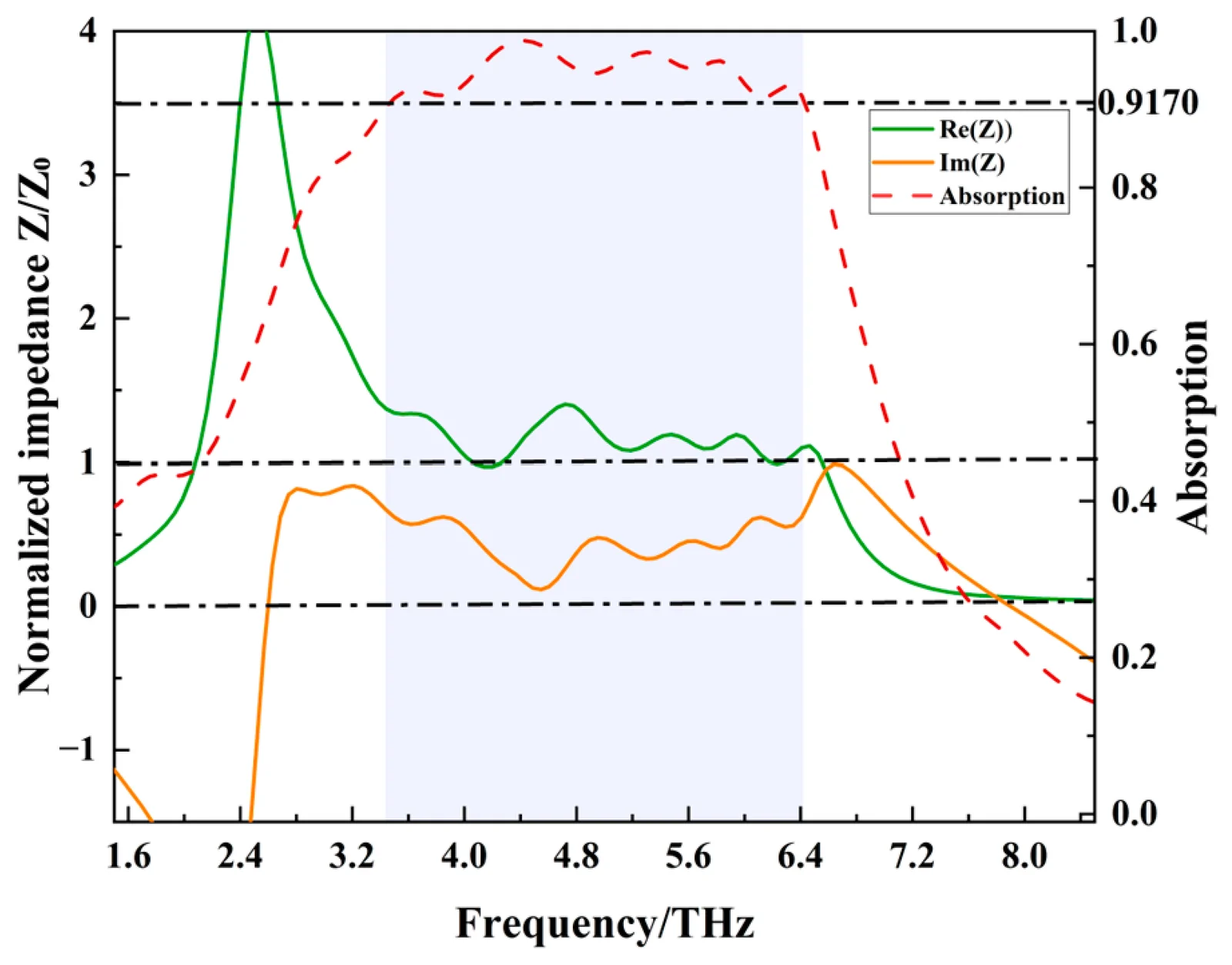
Normalized impedance of the proposed absorber.

Figure 3 shows the normalized impedance of the graphene/MoS_2_ vertical heterojunction-VO_2_ composite absorber.

From the figure, it can be seen that within the range of 3.5–6.5 THz, the real part of the impedance Re[Z(ω)] remains between 0.94 and 1.35. At around 4.14, 5.20, and 6.17 THz, Re[Z(ω)] is close to 1 (for example, at 4.14 THz it is 0.969), indicating that the equivalent resistance is close to the free-space impedance. At around 2.5 THz, Re[Z(ω)] increases to 4.18, and the impedance matching deteriorates, leading to enhanced reflection. The imaginary part of the impedance Im[Z(ω)] changes from negative to positive with frequency.

Within 1–2.56 THz, Im[Z(ω)] ranges from −0.74 to −2.76, which is capacitive and mainly originates from the intraband Drude response of graphene and interlayer capacitance. Around 2.62 THz, it crosses zero. Within 2.74–6.70 THz, Im[Z(ω)] is positive, with a peak of 0.99 at 6.64 THz, related to the inductive response after the VO_2_ phase transition. Above 7.86 THz, Im[Z(ω)] becomes negative again, and at 8.5 THz it is −0.39. By calculating *R* = |(Z − 1)/(Z + 1)|^2^, it can be seen that the reflectivity within 3.5–6.5 THz is less than 8.3%. At 4.37, 5.30, and 6.35 THz, the reflectivity is 1.3%, 2.7%, and 7.1%, respectively; at 4.60 THz, the reflectivity is 0.6%, corresponding to an absorption of 98.7%, which is consistent with the FDTD simulation results.

#### 3.1.3. Quantitative Analysis Based on CMT

To analyze the sources of broadband absorption, the time-domain CMT was employed to model the absorber. FDTD can provide the electromagnetic response, but it is difficult to quantitatively describe the interaction between resonant modes. CMT is mainly used for:

(1) Decomposing broadband absorption into multiple local resonant modes and providing resonant frequencies and linewidths;

(2) Extracting the radiation loss rate $\gamma_{r}$ and absorption loss rate $\gamma_{a}$, and determining the coupling state of each mode;

(3) Analyzing the contribution of multi-mode coherent interference to broadband absorption.

This analysis can be used to explain the physical mechanism of multi-mode broadband absorption and provides quantitative theoretical support for the design of multi-mode hybrid broadband devices based on single-layer two-dimensional materials.

It should be particularly noted that all three modes are under-coupled, with single-mode maximum absorptions of 76.6%, 76.3%, and 70.7%, respectively. However, the total absorption after multi-mode superposition can reach 98.7%. This does not violate energy conservation; it is a typical feature of multi-mode interference. For multi-mode CMT of a single-port reflection system, the total reflection coefficient is the coherent superposition of the complex reflection amplitudes of each mode:

(7) $$r\left(\omega \right)=-1+\sum_{j}\frac{2\gamma_{r,j}}{i\left(\omega -\omega_{0,j}\right)+\gamma_{r,j}+\gamma_{a,j}}$$

When $\gamma_{r}=\gamma_{a}$, the system reaches the critical coupling state, and the single-mode absorption is maximized.

The fitting results show that the near-field coupling between the VO_2_ phase transition layer and the heterojunction unit shifts the intrinsic frequency. The offset of Mode 1 is approximately 13.6% (intrinsic 3.178 THz, observed 3.61 THz), which may be related to the simultaneous influence of the graphene/MoS_2_ heterojunction and the metallic state of VO_2_ on this mode.

**Figure 4:**
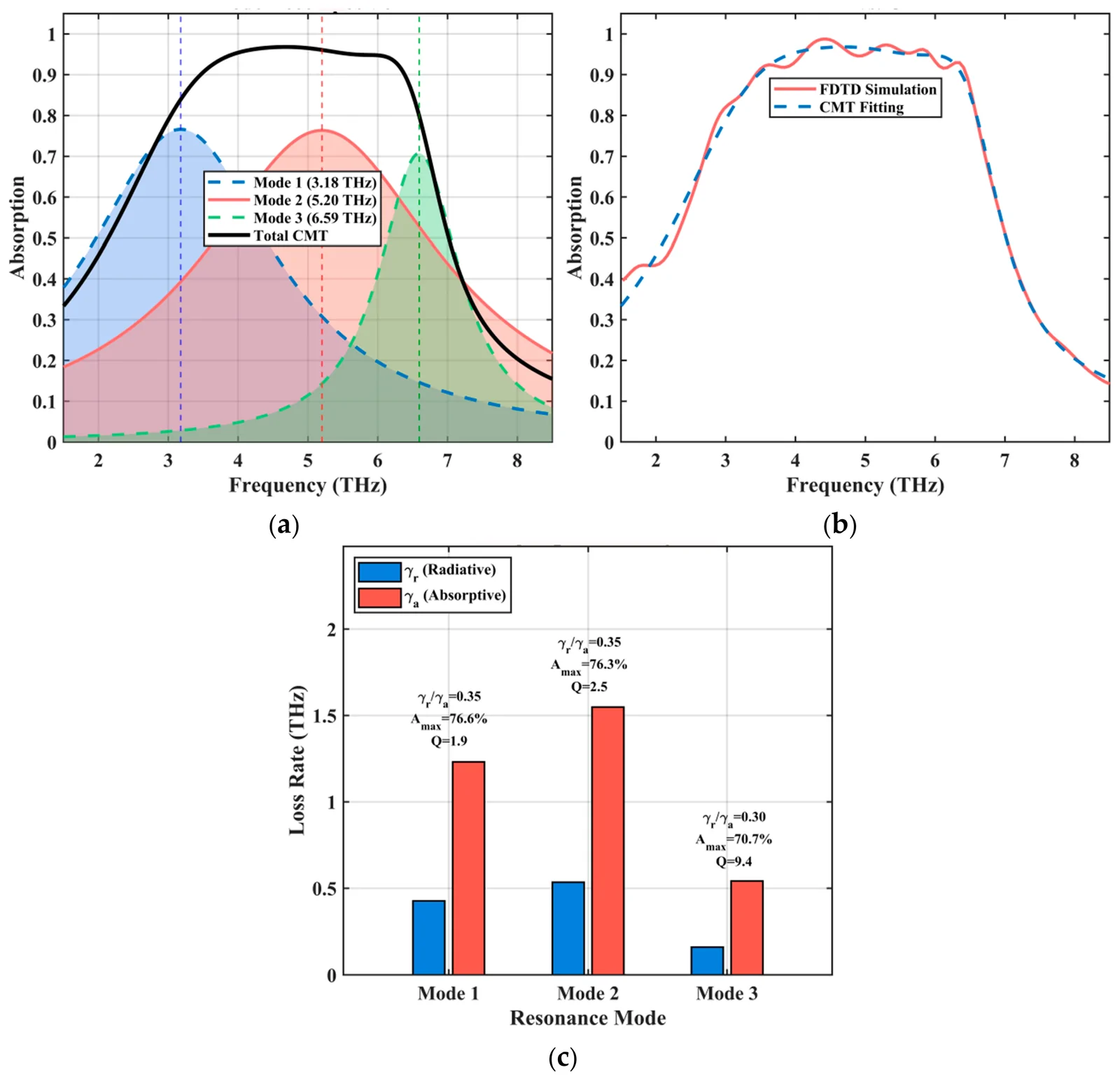
Coupling mode theoretical analysis: (**a**) mode decomposition; (**b**) comparison of FDTD and CMT fitting; (**c**) energy distribution ratio of radiation loss and absorption loss for each mode.

Figure 4a presents the decomposition results of the three modes, and Figure 4b is a comparison of CMT and FDTD, with *R*^2^ = 0.9948. The fitting deviation may come from two aspects: one is the non-Lorentz relaxation during the VO_2_ phase transition process; the other is the nonlinear coupling between the modes in the three-dimensional asymmetric structure, and the linear superposition model cannot fully describe it. All three modes are under-coupled, and the maximum absorption of a single mode is 76.6%, 76.3%, and 70.7%, respectively. In the multimode CMT, the total reflection coefficient is the superposition of the complex reflection amplitudes of each mode, so the total absorption can exceed the upper limit of a single mode. The absorption at 4.37 THz is 98.7%, which is higher than the upper limit of a single mode for Mode 1 and Mode 2, indicating that multimode interference contributes to broadband absorption.

In Table 2, the coupling ratios $\gamma_{r}/\gamma_{a}$ of the three modes are all less than 1, indicating that all the modes are in an under-coupled state. The single-mode absorption limit is determined by the critical coupling condition, but the total absorption of the multi-mode system is determined by the coherent superposition of the complex reflection amplitudes of each mode. Therefore, the under-coupling of the single mode does not prevent the multi-mode system from achieving nearly perfect absorption at a specific frequency. In this paper, the absorption of 98.7% at 4.37 THz is higher than the single-mode upper limit of Mode 1 and Mode 2, which is the result of multi-mode destructive interference.

Three modes collaboratively constructed a broadband, highly absorbing spectrum through the following ultra-wideband three-anchor bridging strategy:

Mode 1 is located at 3.178 THz and is in a moderately under-coupled state. Its total loss rate $\gamma_{r}+\gamma_{a}=1.66$ THz, with Q = 1.91 and FWHM = 3.32 THz. The low-frequency absorption mainly comes from Drude loss within the graphene band, interlayer phonon polariton dissipation in MoS_2_, and metal-state VO_2_ plasmon damping. $\gamma_{a}=1.23$ THz, $\gamma_{r}=0.43$ THz, and the absorption loss account for a large proportion.

Mode 2 is located at 5.200 THz, and its coupling characteristics are similar to Mode 1: $\gamma_{r}/\gamma_{a}=0.345$, Q = 2.50, and FWHM = 4.17 THz. Modes 1 and 2 may correspond to different eigenmodes of the same composite resonator, and the difference in coupling ratio is less than 1%, indicating that their radiation channels and absorption mechanisms are similar. The superposition of the two results in an absorption of over 92% in the 4–5 THz range.

Mode 3 (6.594 THz, high-frequency narrowband precise anchoring) exhibits physical characteristics distinct from the previous two modes: $\gamma_{r}=0.16$ THz drops to 30% of Mode 2′s value, $\gamma_{a}=0.54$ Hz is only 35% of Mode 2′s value, the coupling ratio $\gamma_{r}/\gamma_{a}=0.298$ drops to the lowest level of the three modes, and the quality factor Q = 9.39 increases to 3.8 times that of Mode 2, corresponding to a narrow linewidth FWHM = 1.40 THz. Mode 3 shows low coupling, as well as high Q and narrowband characteristics, and its mechanism is different from the previous two modes.

A detailed analysis reveals that Mode 3 is not a basic dipole resonance but mainly stems from the high-order localized surface plasmon resonances (high-order LSPR) excited by specially sized units (such as units 1, 3, and 6) in the heterojunction. Due to the extremely short resonant wavelength of this mode, the electric field is tightly confined in the nanoscale gaps of the two-dimensional interface, resulting in an extremely small effective radiation cross-section in free space. At the same time, the strong localized high-frequency electric field of this high-order mode undergoes intense intraband high-frequency scattering with the free carriers in the two-dimensional material, providing sufficient non-radiative dissipation (absorption loss). With its high Q value, Mode 3 generates a steep phase jump in the 6.3–7.0 THz frequency band and undergoes constructive interference with the high-frequency tail of Mode 2, precisely compensating for the absorption valley near 6.5 THz and achieving the critical function switching from bridging to anchoring.

The comparison of CMT and FDTD in Figure 4b shows that they match well within 3.5–6.5 THz, but there is a certain deviation above 6.5 THz. The causes of the deviation include: (1) non-Lorentz relaxation during the VO_2_ phase transition; (2) nonlinear coupling between modes in the three-dimensional asymmetric structure; (3) the CMT model does not consider the contribution of high-order modes. Nevertheless, *R*^2^ = 0.9948 indicates that the three-mode CMT model can well describe the main physical mechanism of broadband absorption.

Figure 4c presents the energy distribution weights of each mode in a grouped bar chart, providing an intuitive quantitative representation. The three modes exhibit a trend from moderate under-coupling to deep under-coupling.

This trend is consistent with the loss distribution in the frequency domain of the three material systems. In the low-frequency band (Mode 1), graphene–VO_2_ is dominated by metallic properties, and the radiation channel is relatively open. In the mid-frequency band (Mode 2), the heterojunction–VO_2_ exhibits cooperative resonance, achieving a dynamic balance between radiation and absorption. In the high-frequency band (Mode 3), MoS_2_ is dominated by intraband free carriers, and the two-dimensional confinement effect closes the radiation channel. It is worth emphasizing that although all three modes are in under-coupling states, there are certain differences in coupling ratios. This wide range provides rich degrees of freedom for frequency-domain control. By electrically tuning the graphene $E_{F}$, Mode 1′s $\gamma_{r}$ can be selectively enhanced (increasing the Drude weight towards the radiation channel). By thermally tuning the VO_2_ phase transition, Mode 1 and Mode 2 can be simultaneously regulated $\gamma_{a}$ (the insulating state → metallic state increases the absorption loss by 3–5 times), while Mode 3, as the intrinsic exciton state of MoS_2_, is relatively insensitive to external field control and can serve as a frequency reference anchor. This hybrid strategy of dual tuning and single anchoring is the physical basis for achieving broadband reconfigurable absorption.

### 3.2. Parameter Optimization and In-Depth Exploration of Physical Mechanisms

#### 3.2.1. Unit Contribution Analysis of the Tri-Material Asymmetric Structure

**Figure 5:**
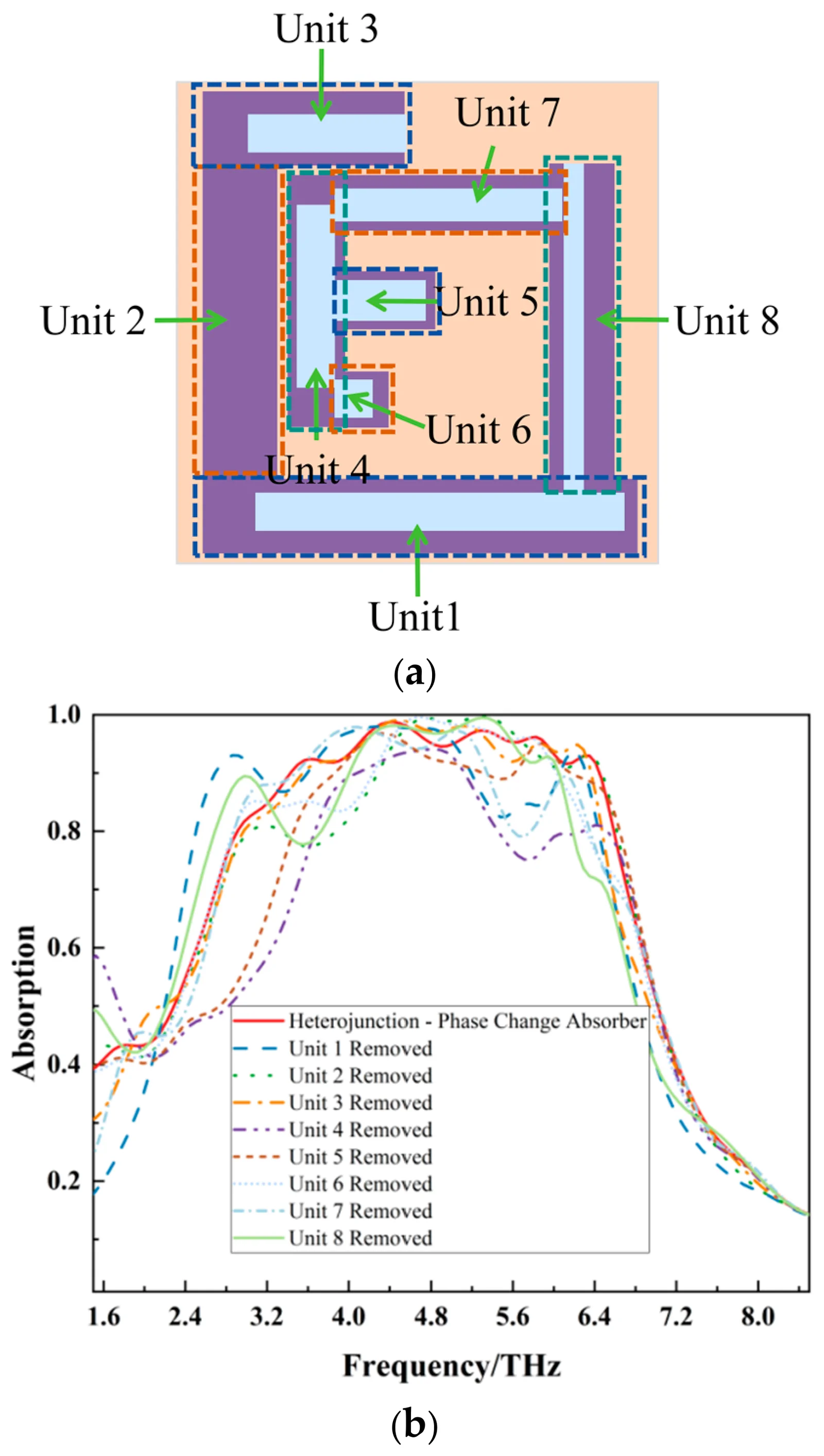

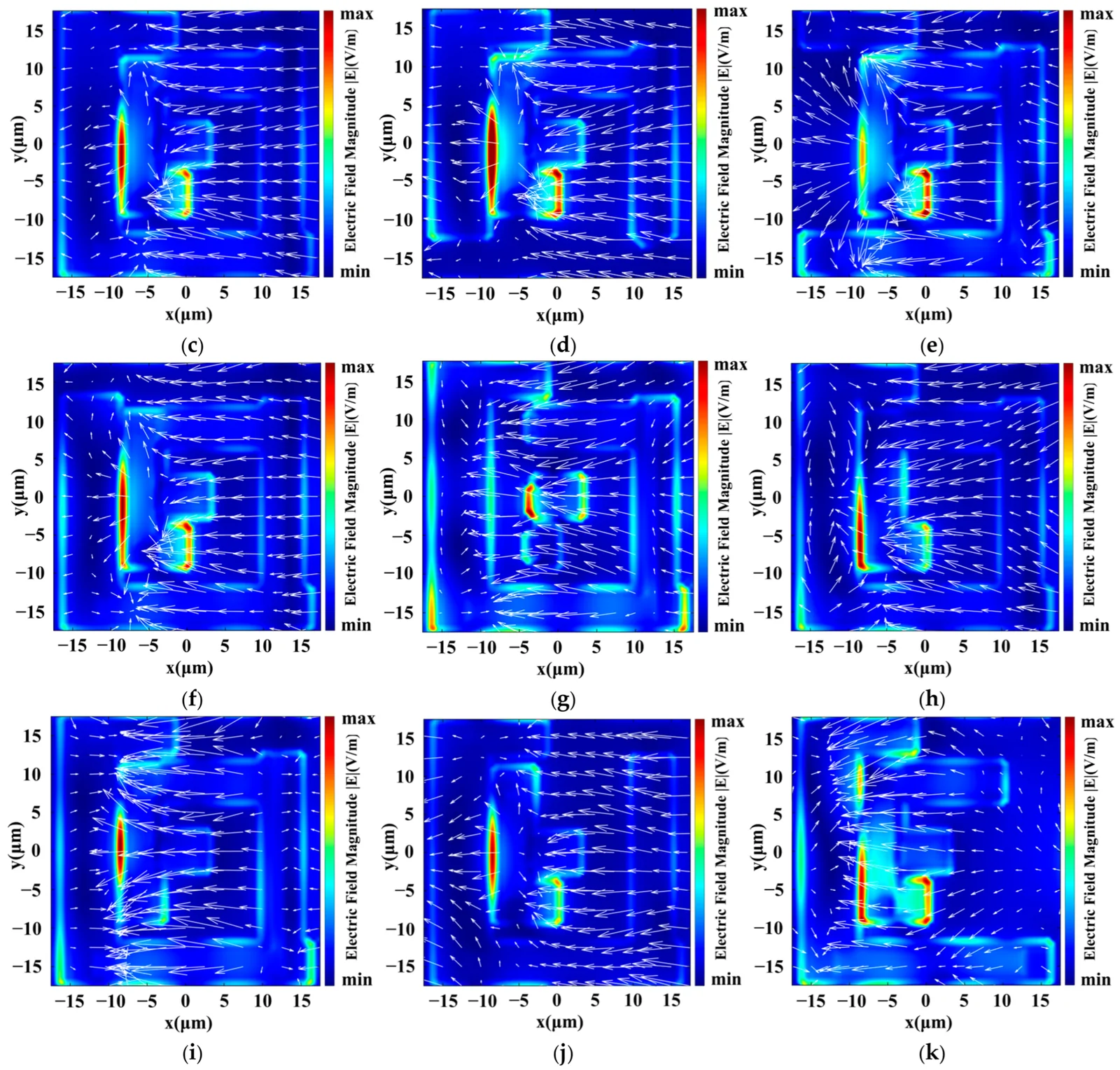
Structural dissection of the absorber and analysis of contributions of each unit: (**a**) top view of the complete structure; (**b**) comparison chart of the complete structure and the absorption spectra after sequentially removing units 1–8; (**c**–**k**) the electric field and current distributions at their respective absorption peak frequencies for the basic structure and the structures after removal of unit 1, unit 2, unit 3, unit 4, unit 5, unit 6, unit 7, and unit 8, respectively.

To reveal the physical functions of each functional unit, this paper sequentially removes 8 asymmetric units while keeping other structural parameters unchanged. Through FDTD simulation, the absorption spectra and field distributions of the degraded structure are obtained. Figure 5a show the top views of the complete structure, and Figure 5b presents the corresponding absorption spectra comparison. Figure 5c–k show the electric field and current distributions at the key frequencies.

Figure 5b compares the absorption spectra of the intact absorber and degraded structures after sequentially removing each unit. The complete structure achieves continuous broadband absorption exceeding 90% across 3.5–6.5 THz (peaking at 98.7% near 4.4 THz). Omitting any single unit leads to bandwidth contraction, peak decay, or flatness deterioration to varying degrees. Based on these spectral degradation signatures, the physical roles of the eight asymmetric units can be categorized into three distinct mechanisms:

(1) Units 2, 6, and 8: The main support for low-frequency absorption. Removing units 2 or 8 would reduce the absorption in the 3.2–4.0 THz frequency band from over 90% to 77–82%; removing unit 6 would also lower the absorption in this frequency band to below 85%. These units mainly excite low-frequency localized surface plasmon resonances and are strongly coupled to the underlying MoS_2_, providing sufficient low-frequency ohmic loss, corresponding to the low-frequency anchoring mode (Mode 1) in the CMT theory. Among them, unit 8 has the largest equivalent resonant size, and the long-wavelength dipole resonance it excites is the foundation of the entire low-frequency absorption platform; units 2 and 6 enhance low-frequency energy convergence through near-field coupling.

(2) Units 1, 4, and 7: Key contributions to high-frequency absorption expansion. Removing unit 4 would cause the absorption at 6.0 THz to drop sharply from 93.2% to 79.0%, significantly shrinking the high-frequency bandwidth boundary; removing unit 1 or 7 would reduce the absorption in the 5.2–6.5 THz frequency band to below 90%. These smaller-sized units, excited by higher-order dipole resonances, dominate the high-frequency resonance, corresponding to the high-frequency precise anchoring mode (Mode 3) in the CMT theory. They not only capture high-frequency energy themselves but also suppress the high-frequency scattering of adjacent units through near-field interactions, thereby stabilizing the perfect absorption boundary at 6.5 THz. Among them, unit 4 has the most significant contribution to the high-frequency expansion, while units 1 and 7 play an auxiliary enhancing role.

(3) Units 3 and 5: Removing either of these two units will not cause the complete collapse of broadband absorption, but it will significantly reduce the flatness of the absorption spectrum. Removing unit 5 will make the transition edge in the 3.5–4.0 THz region less steep. Removing unit 3 will cause the absorption in the high-frequency band to decrease by approximately 3.0% and in the low-frequency band by approximately 4.3%. These units achieve vector coherent cancelation with the spectral tails of the high and low-frequency main resonant units through the phase evolution of the local excitation field, effectively filling the absorption valleys between different characteristic frequencies, corresponding to the bridging compensation mechanism of Mode 2 in the CMT theory. Their functions can be partially compensated by the enhanced coupling parts of adjacent units, so the performance degradation after removal is relatively small.

These results indicate that the broadband absorption of the graphene/MoS_2_–VO_2_ asymmetric structure does not arise from a linear superposition of individual unit contributions, but from near-field nonlinear coupling and current-topology reconstruction among the eight units. As shown in Figure 5c–k, the intact structure exhibits a dominant right-to-left current flow converging at the gaps between Units 2, 4, and 6, with electric-field hotspots concentrated at the Unit 2–4 gap and the edges of Unit 6—forming the microscopic basis for the coexistence and synergy of multiple resonant modes. Removing any core unit triggers a global reconstruction of the current paths (detouring, reversal, or branching) and redistribution of field hotspots, but none of these reconfigurations fully restores the impedance-matching efficiency of the original topology. This confirms that the precise asymmetric arrangement of the eight units is essential for achieving simultaneous mode broadening and impedance matching across the full operating band.

#### 3.2.2. Analysis of the Synergistic Loss Mechanism Among Three Materials

To analyze the individual roles of graphene, MoS_2_, and VO_2_, five structural configurations are compared. The synergy among the three materials is not a linear superposition of their individual absorption cross-sections, but a collaborative effect based on frequency-domain division of labor and spatial complementarity: VO_2_ sets the global impedance boundary of the longitudinal cavity, graphene provides a continuously tunable in-plane current channel, and MoS_2_ governs the intensity and distribution of local plasmon resonance.

**Figure 6:**
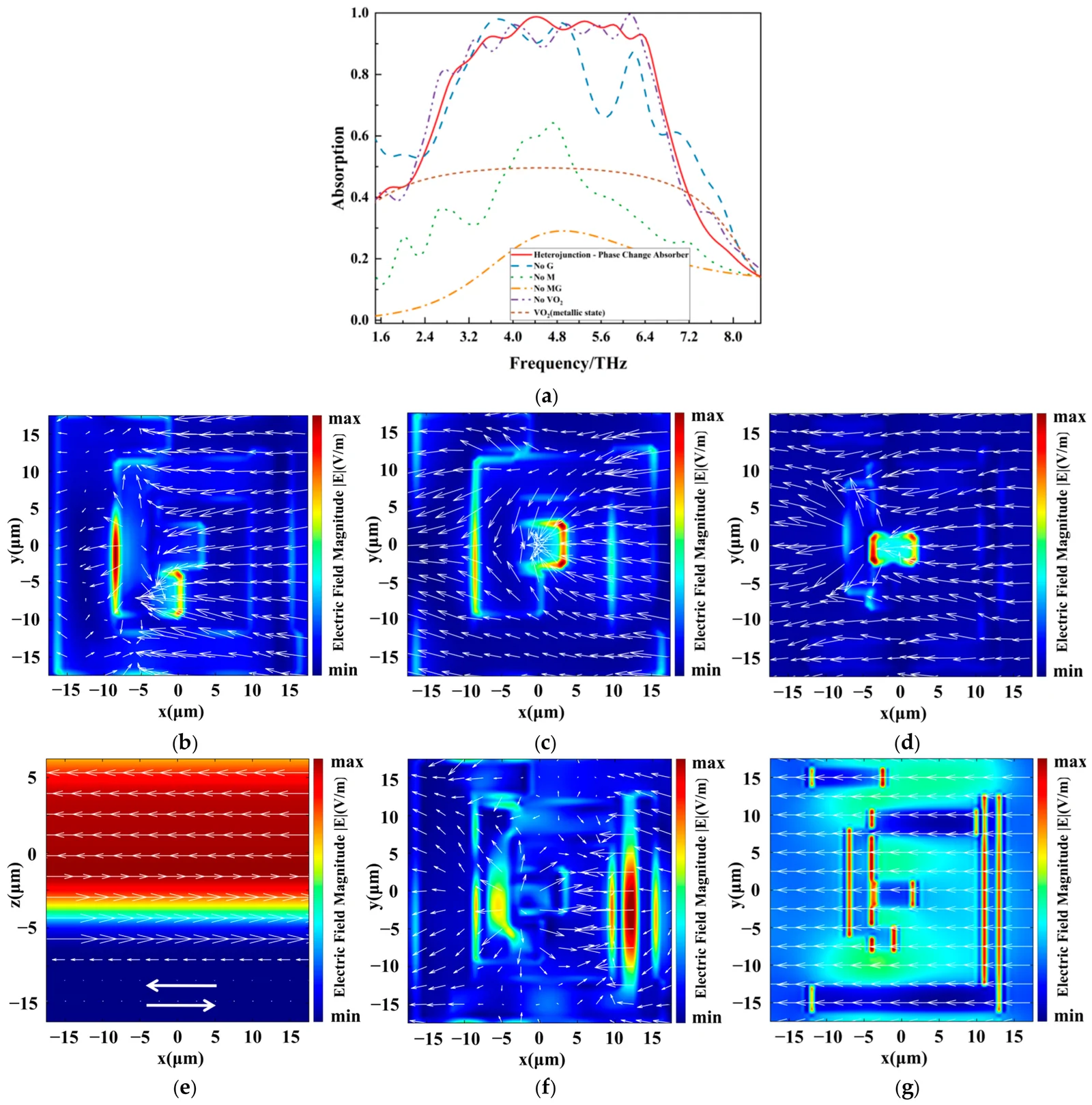

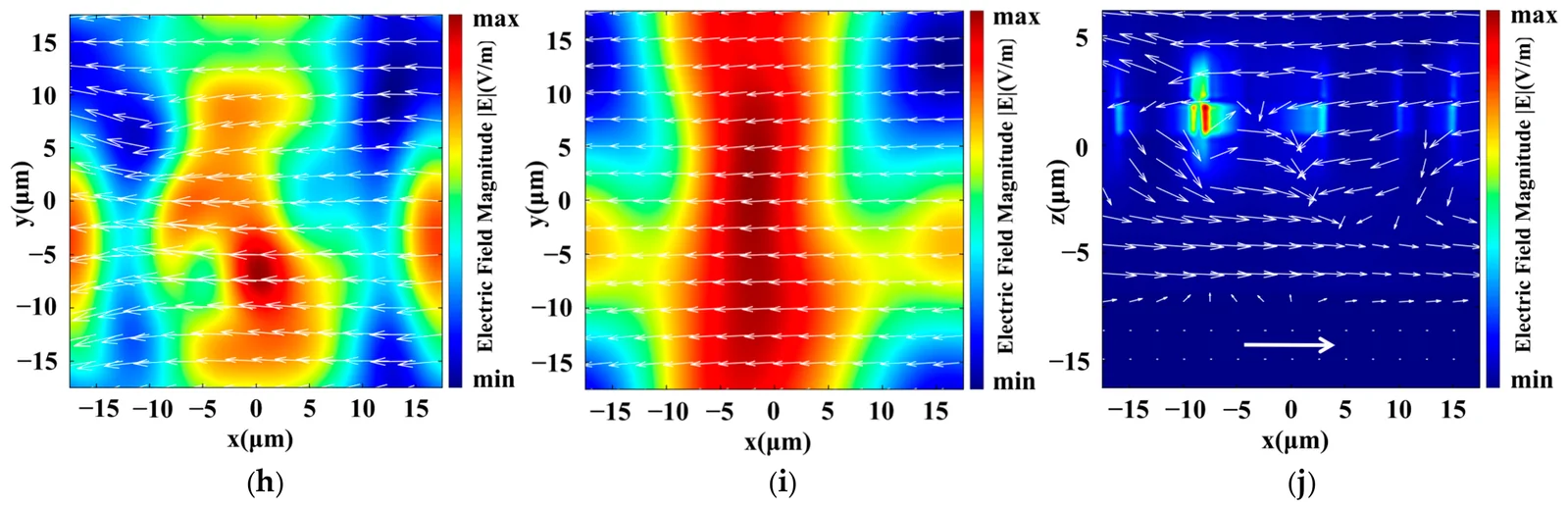
Material synergy analysis and electromagnetic field characterization of the absorber. (**a**) Absorption spectra under six material configurations: complete heterojunction, without graphene patterned layer, without MoS_2_ patterned layer, and structures with only vanadium dioxide/PI dielectric layer/gold reflector layer, without vanadium dioxide layer, and vanadium dioxide in metallic state; (**b**) electric field and surface current distributions for the complete structure; (**c**) electric field and current distributions for the structure without graphene patterned layer; (**d**) electric field and current distributions for the structure without MoS_2_ patterned layer; (**e**) electric field and current distributions for the structure with only vanadium dioxide/PI dielectric layer/gold reflector layer; (**f**) electric field and current distributions for the structure without vanadium dioxide layer; (**g**) electric field and current distributions when vanadium dioxide is in metallic state; (**h**) electric field and current distributions in the PI dielectric layer of the basic structure; (**i**) electric field and current distributions in the bottom gold reflector layer of the basic structure; (**j**) electric field and current distributions in the longitudinal X-Z cross-section of the basic structure.

(1) Absorption complementarity in the 2D heterojunction. Removing both graphene and MoS_2_ reduces the full-band absorption to only 29.0% (~4.89 THz), confirming that PI and insulating-state VO_2_ contribute negligible loss and that absorption mainly originates from the top 2D pattern. Removing only MoS_2_ broadens the spectrum but lowers the peak to 64.2% (~4.72 THz), since graphene’s broadband Drude loss alone provides insufficient local field strength for perfect impedance matching. Removing only graphene raises the peak to 98.0% (3.73 THz) and 96.7% (4.89 THz), but sharpens the resonance, with absorption falling below 90% outside 3.3–5.2 THz. When combined, MoS_2_′s strong local plasmon resonance enhances the absorption peak while graphene’s broadband free-carrier scattering widens the linewidth; interfacial charge transfer further raises the effective conductivity, jointly enabling continuous high absorption across 3.5–6.5 THz.

(2) Dynamic impedance reconfiguration via VO_2_ phase transition. Removing the insulating-state VO_2_ layer (with MoS_2_ shifted down by 0.17 μm) still yields > 90% absorption over 3.2–6.3 THz (peak 99.6% at 6.11 THz), indicating that insulating VO_2_ mainly acts as a high-permittivity matching layer that fine-tunes the Fabry–Perot cavity’s effective optical thickness and interfacial phase delay, smoothing the spacing between adjacent resonant modes; without it, band flatness and overall absorption slightly deteriorate. In contrast, when VO_2_ transitions to the metallic state, absorption collapses below 50% across the entire 1.0–8.5 THz range: the highly conductive VO_2_ film acts as an electromagnetic shield above the PI cavity, disrupting the Fabry–Perot resonance condition and causing severe impedance mismatch, so most incident energy is reflected at the top surface—yielding a macroscopic absorption/reflection switching modulation depth exceeding 50%.

(3) Microscopic field and current distribution. Figure 6b–g reveal the loss mechanism of each material. In the complete structure, the electric field is strongly localized at the gap between Units 2 and 4, with current converging at multiple unit gaps along a dominant right-to-left channel, confirming that multi-unit near-field coupling underlies broadband absorption. Removing graphene redirects the residual field/current toward Unit 5, as the system loses its broadband Drude-loss channel and seeks a new low-resistance path—confirming graphene’s role in dispersing energy across multiple units to widen the absorption bandwidth. Removing MoS_2_ weakens the overall field intensity but still shows partial convergence at Unit 5, confirming that MoS_2_ mainly provides strong local plasmon enhancement of the absorption peak rather than bandwidth broadening. For the bare VO_2_/PI/Au structure without any 2D pattern, the field and current appear only in the x–z cross-section as a weak reverse-parallel pair (skin-effect/image-current response), with absorption limited to <29%, confirming that the top heterojunction pattern is an essential prerequisite for strong impedance matching. Removing VO_2_ shifts the resonant band (while preserving >90% absorption) due to the altered cavity phase delay, with Unit 8—the largest unit—being most sensitive to this shift. When VO_2_ is in the metallic state, the field collapses from a multi-hotspot pattern to simple edge-dipole hotspots on individual graphene strips, with unidirectional parallel current and no closed-loop path, reflecting the loss of the longitudinal resonant cavity and confirming that the insulating-state VO_2_ layer serves as an indispensable transparent cavity window for broadband absorption.

(4) Longitudinal energy coupling and magnetic resonance. The field distributions in the PI layer and the Au ground plane [Figure 6h,i] spatially mirror the top pattern, with enhancement near Units 4–6, and the displacement/induced currents flow opposite to the top-layer current—evidence that near-field energy excited by the asymmetric top pattern penetrates vertically through the PI spacer and induces currents in the gold layer, forming a closed cross-layer current loop. The x–z cross-section [Figure 6j] confirms this: the top-layer current (flowing right-to-left) reverses direction in the VO_2_/PI/Au stack below (flowing left-to-right), and part of the top current flows downward into the VO_2_ layer before redirecting horizontally—demonstrating that the insulating-state VO_2_ layer actively participates in, rather than passively blocks, longitudinal electromagnetic energy transfer. This closed vertical current loop generates a magnetic dipole moment perpendicular to the structure plane, providing an efficient longitudinal energy-capture and dissipation channel for broadband absorption, with the strongest field-current coupling occurring at the Unit 2–4 gap—fully consistent with their identification as core units in the structural dissection analysis.

In summary, these three materials are not simply stacked. Graphene and MoS_2_ provide broadband absorption through Drude broadband response and local plasmon resonance, while VO_2_ changes impedance through the insulation-metal transition to achieve a modulation depth of approximately 50%. Through interfacial charge transfer and near-field coupling, the three materials divide low-, mid-, and high-frequency energy-dissipation tasks in a complementary manner.

#### 3.2.3. Influence of Key Structural Parameters

Influence of PI Dielectric Layer Thickness

**Figure 7:**
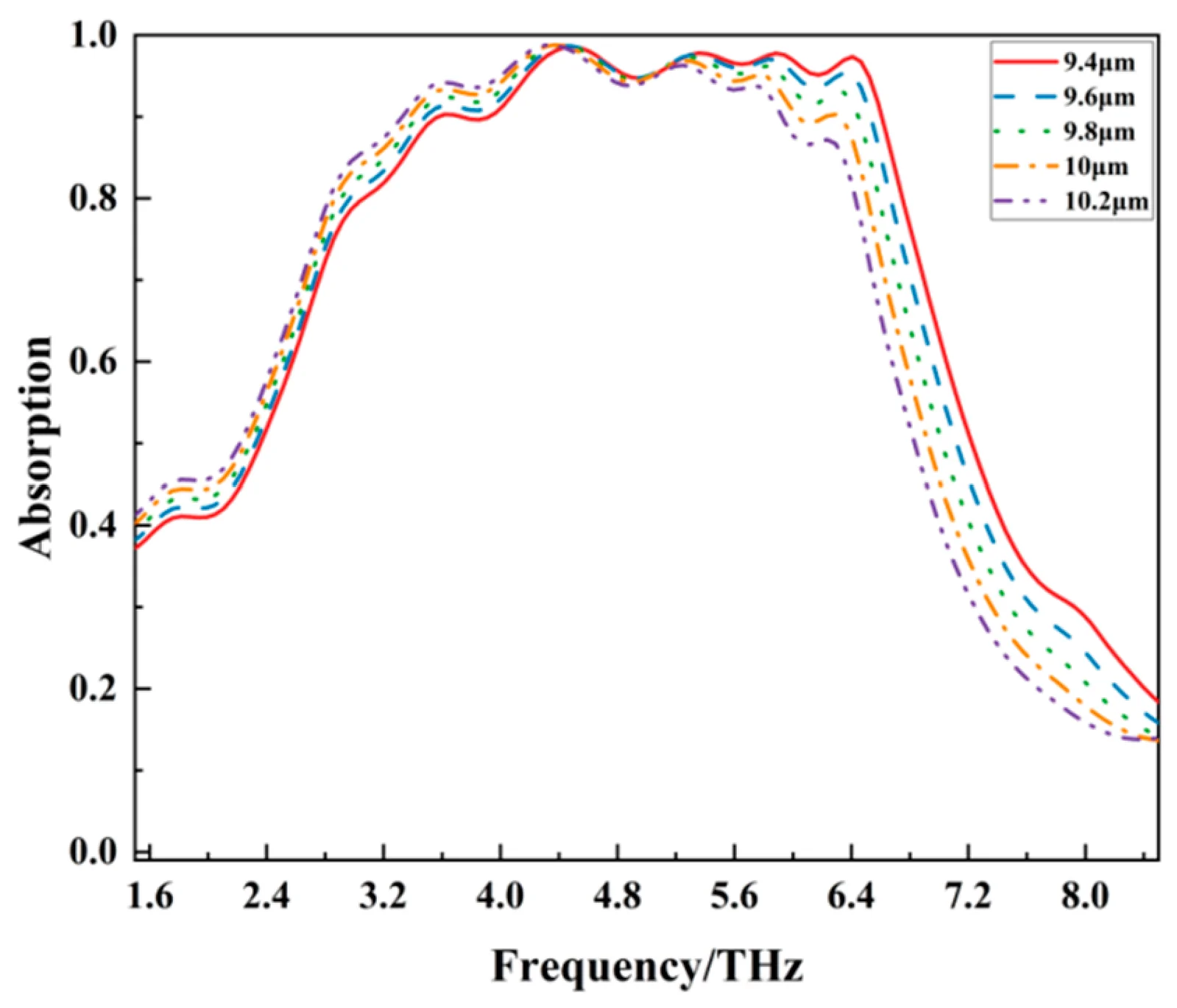
Influence of the thickness of the dielectric layer h1 on the absorption characteristic.

The PI dielectric layer (thickness h1) serves both as a physical spacer between the top heterostructure and the bottom reflector and as the core optical cavity of the asymmetric Fabry–Perot resonator. This section analyzes five thickness values (h1 = 9.4, 9.6, 9.8, 10.0, 10.2 μm); results are shown in Figure 7.

As the h1 value increases, the absorption spectrum mainly undergoes the following changes:

(1) Redshift of the resonant frequency with increasing h1. As h1 increases from 9.4 to 10.2 μm, the absorption spectrum shows a systematic redshift across the band; for example, the low-frequency resonant peak shifts from ~4.49 THz (98.57%, h1 = 9.4 μm) to ~4.43 THz (98.75%, h1 = 9.8 μm) and further to ~4.31 THz (h1 = 10.2 μm). This follows directly from the Fabry–Perot phase-matching condition, under which a larger cavity thickness requires a proportionally lower resonant frequency to maintain constant round-trip phase.

(2) Evolution of three-mode coupling and bandwidth flatness. The broadband platform relies on the superposition of three under-coupled modes near 4.4, 5.3, and 6.3 THz, whose optimal impedance matching shifts with h1. At h1 = 9.4 μm, the high-frequency mode is well matched (97.33% at 6.40 THz; >90% band extends to 6.57 THz), but the low-frequency edge contracts. At h1 = 10.2 μm, the low- and mid-frequency modes remain well matched (98.76% at 4.31 THz; 96.30% at 5.24 THz), but high-frequency matching deteriorates sharply (87.68% at 6.0 THz; 81.53% at 6.40 THz), since the overly thick cavity pushes the high-frequency spatial impedance away from free-space impedance. The absorption valley near 4.9 THz (between the first two modes) also deepens slightly with increasing thickness (94.71% → 93.79%), reflecting weakened inter-mode compensation as the modal spacing widens. Based on this comparison, h1 = 9.8 μm is selected as the standard value, achieving the best overall three-mode balance: a peak of 98.75% at 4.43 THz and a stable >90% band from 3.44 to 6.45 THz (>3 THz absolute bandwidth).

Manufacturing tolerance analysis of unit gaps

**Figure 8:**
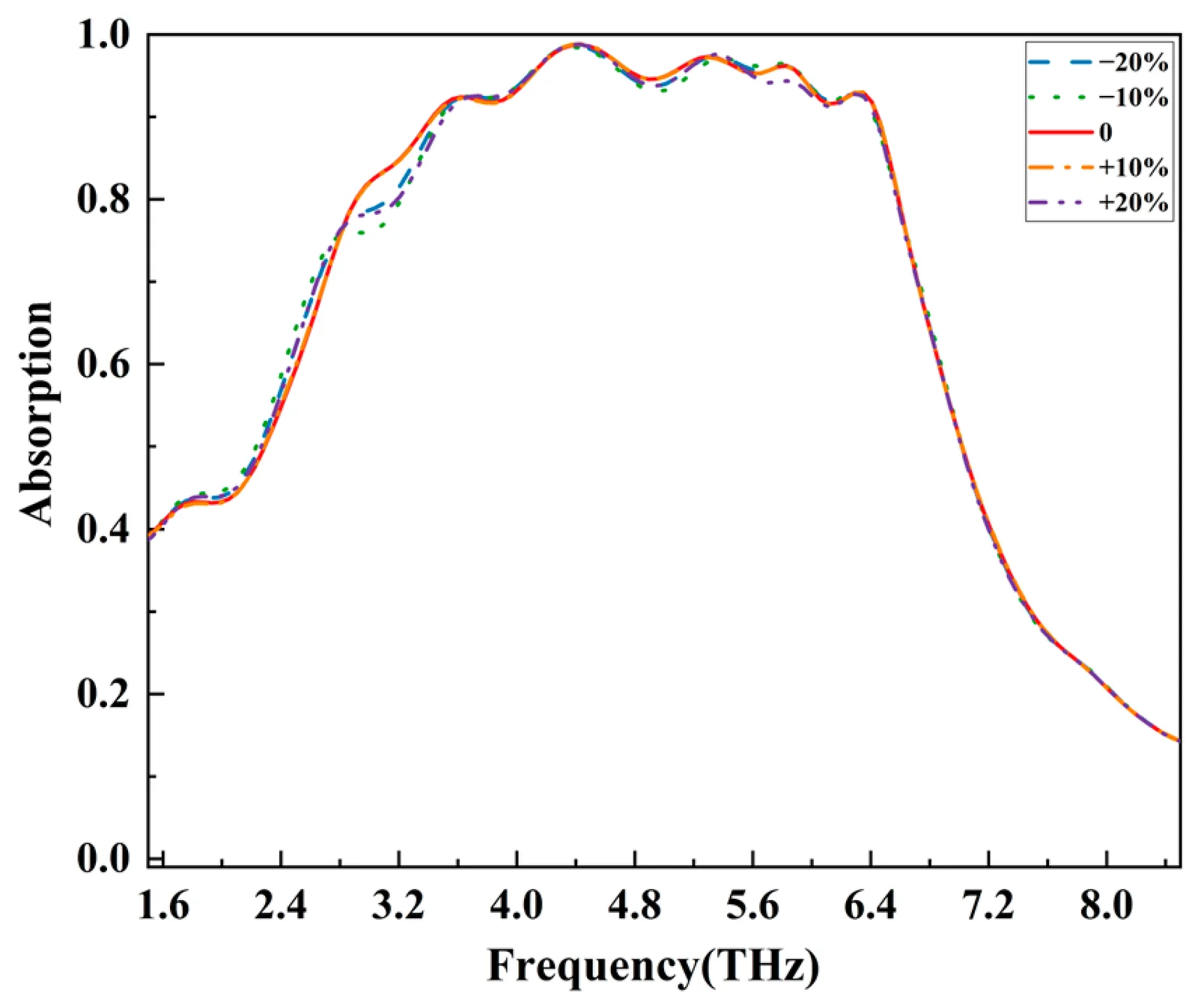
Manufacturing tolerance analysis of the critical parameter (gap between Unit 2 and Unit 4).

In the actual processing of metamaterial devices, the gap size between the patterned units is often limited by factors such as lithography accuracy and etching uniformity. To evaluate the robustness of this three-material composite absorber against manufacturing errors, this section conducts a systematic tolerance analysis of the key gap between unit 2 and unit 4. The designed value of this gap is 1.1 μm. We evaluated its deviation by ±10% and ±20%, resulting in gap sizes of 0.88 μm (−20%), 0.99 μm (−10%), 1.1 μm (0%), 1.21 μm (+10%), and 1.32 μm (+20%), as shown in Figure 8.

Under the standard gap (1.1 μm), the maximum absorption peak is at 4.43 THz, with an absorption rate of 98.75%. When the gap undergoes ±10% and ±20% changes, the absorption peak position remains stable within a narrow range of 4.37 to 4.43 THz, and the highest absorption rate remains at 98.85% (−10%), 98.47% (+10%), 98.60% (−20%), and 98.79% (+20%). The peak fluctuation amplitude is less than 0.4%, demonstrating extremely high amplitude stability.

Taking the wide-band absorption range with an absorption rate greater than 90% as an example, the standard structure covers 3.44 to 6.45 THz (absolute bandwidth 3.01 THz). In the most extreme deviations of −20% (gap reduced to 0.88 μm) and +20% (gap expanded to 1.32 μm), the effective working frequency band greater than 90% becomes 3.47 to 6.44 THz and 3.53 to 6.42 THz, respectively. For both the edge position and the total absolute bandwidth, the deviation from the theoretical value is less than 2%.

These data indicate that this ultra-wideband absorber has extremely strong immunity to the tolerance of internal unit gaps. This is mainly because the ultra-wideband absorption mechanism of this device does not rely on the fragile lateral capacitance coupling (such as slits) between adjacent resonant patches, but is mainly dominated by the longitudinal Fabry–Perot resonance formed by the long and wide geometric dimensions of the top two-dimensional heterostructure and the bottom metal reflector. In addition, the continuous VO_2_ film further smooths the sudden change in the local electromagnetic field. This excellent fabrication tolerance significantly reduces the fabrication difficulty and cost of actual device manufacturing.

In this superstructure, the gap between unit 2 and unit 4 is the core nanoscale gap that is most sensitive to electromagnetic coupling. The deviations of ±10% and ±20% evaluated in our simulation correspond to gap variations of ±0.11 μm and ±0.22 μm, respectively, relative to the nominal 1.1 μm slot width. This sub-wavelength dimensional offset is completely within the achievable range of standard electron beam lithography, which typically can achieve CD uniformity and overlap accuracy better than 50–100 nm. At the same time, this level can also be achieved through advanced deep ultraviolet step lithography technology (with a CD control range of approximately 100–200 nm) used for the fabrication of terahertz superstructures. This indicates that, under the current micro/nano processing technologies, the <2% performance degradation caused by a ±20% gap deviation falls within an easily achievable manufacturing tolerance window.

#### 3.2.4. Synergistic Tuning Characteristics of Three Mechanisms

This section analyzes the regulating effects of VO_2_ phase state, graphene’s $E_{F}$, and MoS_2_ carrier concentration on the absorption spectrum.

(1) Broadband/Narrowband Mode Switching Driven by VO_2_ Phase Transition

A continuous VO_2_ film serves as the impedance control layer. By controlling the temperature across the phase-transition critical point (~68 °C), VO_2_ reversibly switches between insulating and metallic states, enabling significant switching and reconstruction of the terahertz absorption spectrum.

**Figure 9:**
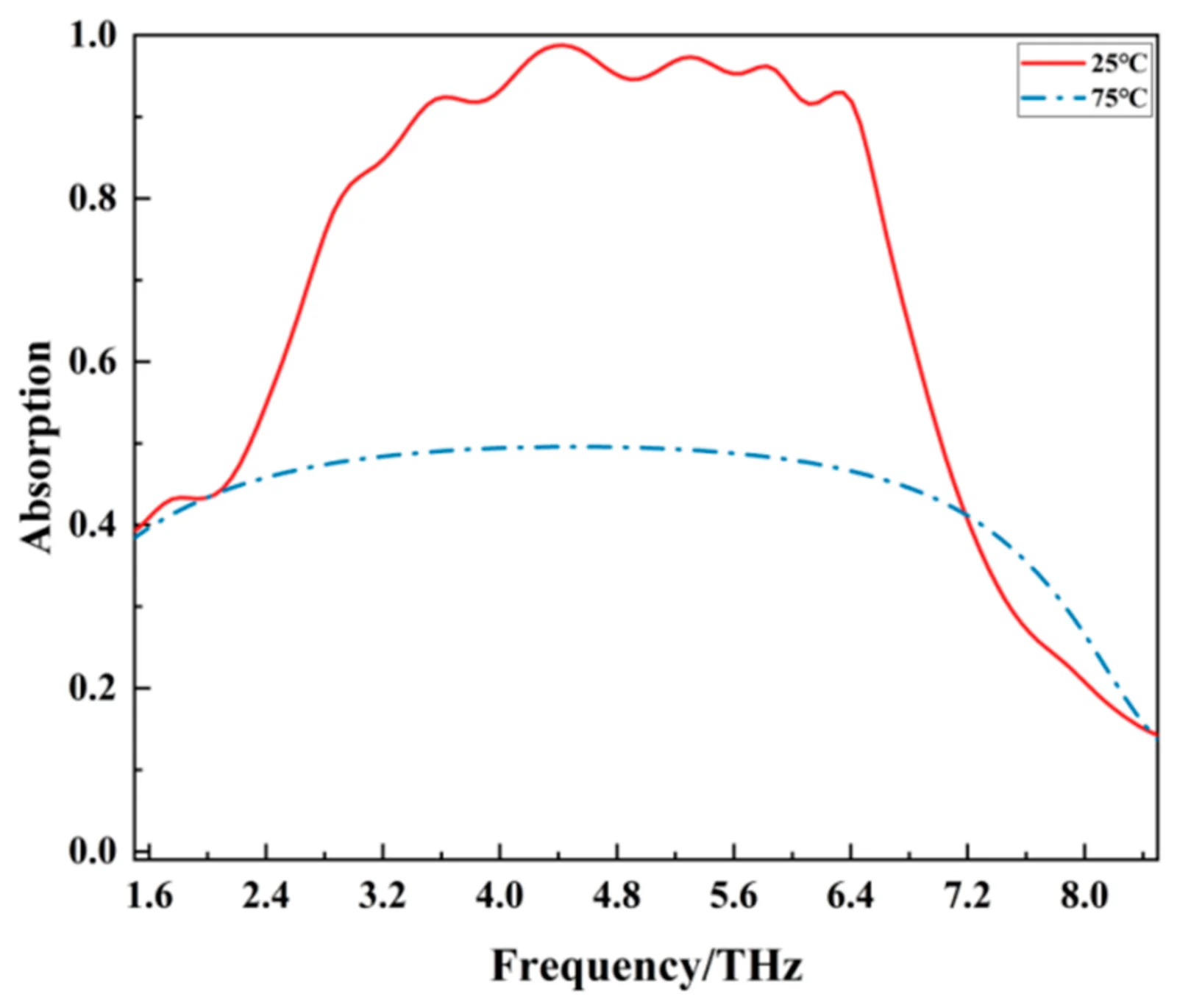
Switching response of the terahertz absorber spectrum driven by VO_2_ phase transition.

Insulating state (wideband high absorption mode). At room temperature (25 °C), as shown in Figure 9, VO_2_ exhibits low conductivity and behaves as a low-loss, high-permittivity medium, allowing incident waves to effectively penetrate into the 9.8-μm PI cavity. The device thus functions as an ultra-wideband F-P resonator with VO_2_ serving as a high-permittivity matching layer, achieving >90% absorption over 3.44–6.45 THz with a near-perfect peak of 98.75% at 4.43 THz.

Metallic state (inefficient absorption/reflection switching mode). When the heating exceeds the phase transition temperature (75 °C), VO_2_ transforms from the monoclinic phase to the rutile phase and enters the metallic state, resulting in an increase in electrical conductivity by approximately three orders of magnitude. This causes a sudden drop in absorption across the entire 1.0–8.5 THz frequency range, falling below 49.5%, and further drops to 47.9% at 6.0 THz and only 13.7% at 8.5 THz. At this point, the high-conductivity VO_2_ film located between the two-dimensional heterojunction and the PI layer acts as a reflector, preventing terahertz waves from entering the underlying dielectric cavity, thereby disrupting the previously carefully designed longitudinal F-P resonant phase-matching conditions. At this time, the device essentially degenerates into a thin graphene/MoS_2_ heterojunction + metal backplane structure without a cavity, with the remaining 40–49% absorption solely coming from the ohmic loss of the top two-dimensional heterojunction itself and the weak local plasmon resonance.

(2) Continuous fine-tuning of graphene’s $E_{F}$

In the VO_2_ insulating state, the device operates in a broadband high absorption mode. This section further analyzes the regulating effect of the graphene $E_{F}$ on impedance matching. By applying an external bias voltage, the $E_{F}$ of graphene is continuously adjusted within the range of 0.2 eV to 0.8 eV. The evolution law of its absorption spectrum reflects the optimization process of the device in terms of impedance matching.

Extracting the absorption spectrum data at different $E_{F}$ allows us to observe the following significant features:

Optimization of Band Broadening and Flattening of Absorption

When $E_{F}$ is at a lower level (such as 0.2 eV), the surface conductivity of graphene is low, and the device exhibits a narrow band and relatively low absorption characteristics (the highest absorption peak is at 4.72 THz, with an absorption of 92.38%). The frequency band with an absorption greater than 90% is relatively narrow, covering only 4.48–5.01 THz.

As $E_{F}$ gradually increases to 0.4 eV and 0.6 eV, the absorption spectrum rapidly widens on both sides, and the overall absorption rate significantly increases. For example, when $E_{F}=0.6$ eV, an extremely high absorption peak of 99.09% is reached at 5.24 THz.

When $E_{F}$ reaches the design standard value of 0.8 eV, although the absolute peak (98.75%) is slightly lower than 0.6 eV, the edge impedance mismatch of the band has been perfectly compensated, and the absorption in the high-frequency part significantly increases, allowing the super-wideband platform with an absorption > 90% to extend stably from 3.44 THz to 6.45 THz. Within the scope of this calculation, when $E_{F}=0.8$ eV, the absorption bandwidth and edge impedance matching performance of the device are relatively good. This is the optimal value in terms of overall performance.

The dynamic blue shift of the absorption peak position

**Figure 10:**
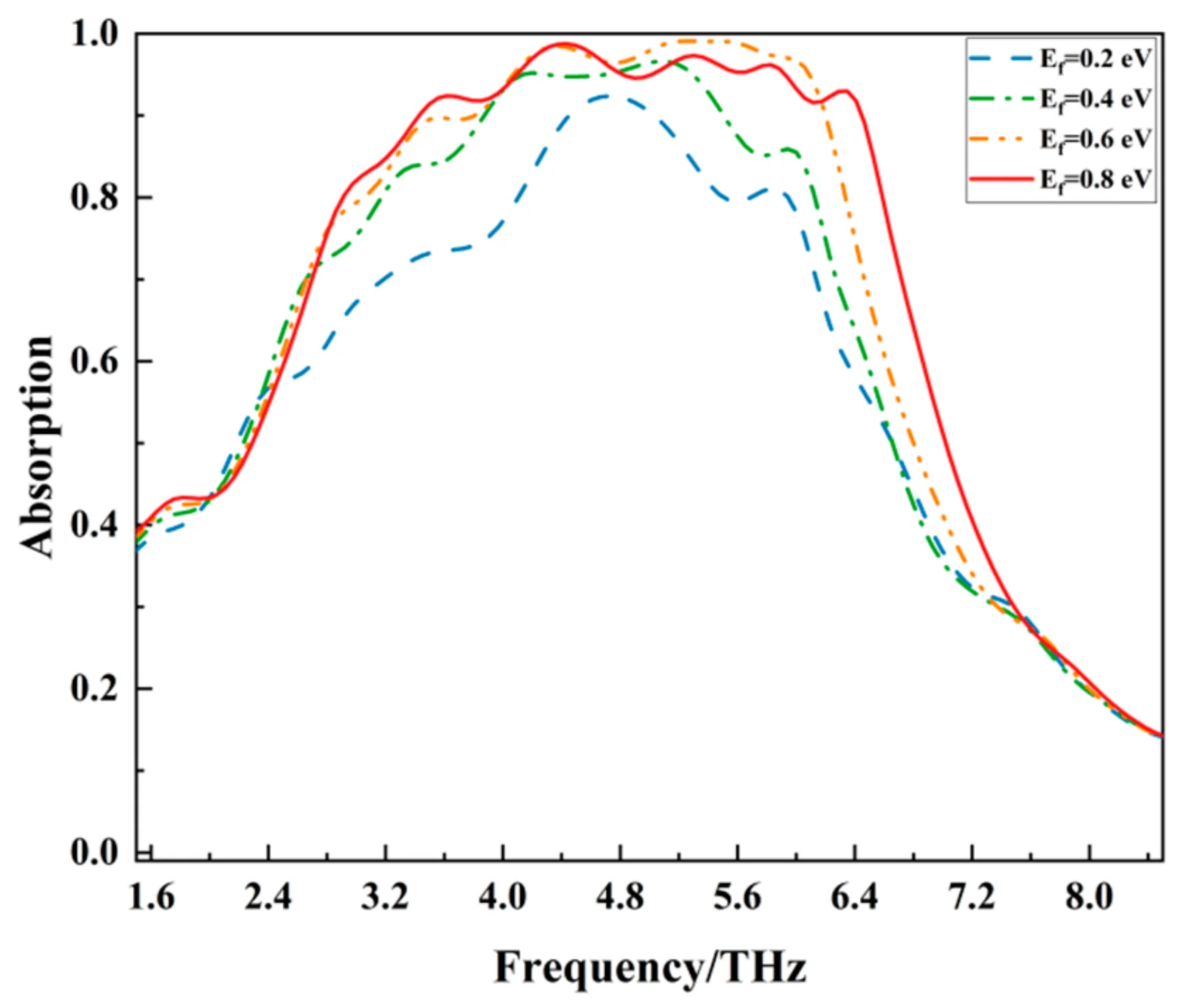
High-frequency blue shift and bandwidth evolution of the absorber under different graphene $E_{F}$.

Figure 10 shows that as the $E_{F}$ of graphene increases, the resonant center frequency undergoes a significant blue shift: 0.2 eV (center approximately 4.7 THz) → 0.4 eV (center approximately 5.06 THz) → 0.6 eV (center approximately 5.24 THz). According to the Drude model of graphene, raising the $E_{F}$ not only increases the intraband transition loss of the system (the real part of conductivity increases, optimizing impedance matching), but more importantly, it changes the kinetic inductance of graphene. Due to $L_{k}\propto 1/E_{F}$, the increase in the $E_{F}$ directly leads to a reduction in the equivalent kinetic inductance of the two-dimensional layer. According to the resonant frequency formula f∝1/LC, the reduction in inductance macroscopically drives the absorption peak to smoothly move towards the high-frequency (blue-shift) direction. Adjusting the $E_{F}$ of graphene can adjust the absorption depth and bandwidth boundary without changing the geometric size.

(3) Auxiliary modulation for carrier concentration control in MoS_2_

Apart from VO_2_ and graphene, MoS_2_ mainly provides low-loss enhancement in the heterojunction. To verify its ability to supplement and regulate the spectrum, VO_2_ (insulating state) and the $E_{F}$ of graphene (0.8 eV) were fixed, and the carrier concentration of MoS_2_ was controlled by using an ionic liquid gate. The ionic liquid gate structure is as follows: an ionic liquid layer is covered on the surface of MoS_2_, and a voltage is applied through the side gate, using the double-layer effect to induce a high density of carriers on the surface of MoS_2_.

**Figure 11:**
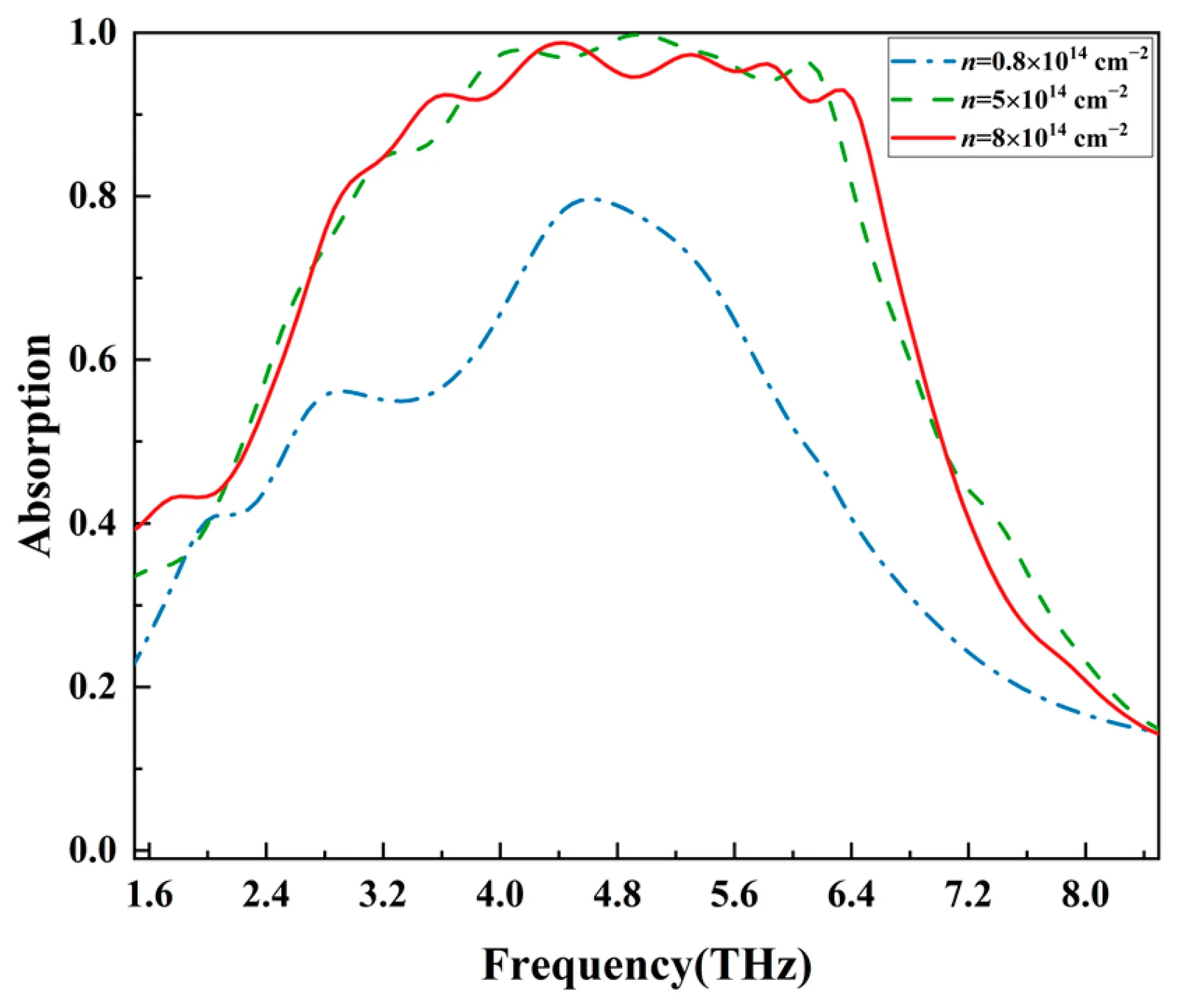
Auxiliary modulation effect of MoS_2_ carrier concentration on terahertz broadband absorption intensity.

Low concentration state ($n=0.8\times 10^{14}$ cm^−2^): When the carrier concentration of MoS_2_ is extremely low, the total equivalent conductivity of the heterojunction is insufficient. As shown in Figure 11, the absorption across the entire terahertz frequency range significantly collapses, with the peak value being only 79.65% (at 4.60 THz), and the broadband perfect absorption characteristic is completely lost.

Medium concentration state ($n=5\times 10^{14}$ cm^−2^): As the carrier concentration increases, the device excites a perfect absorption peak of up to 99.78% at 4.95 THz. However, the absorption in the high-frequency band (>6 THz) declines, and the broadband flatness is insufficient.

High concentration reference state ($n=8\times 10^{14}$ cm^−2^): When the concentration reaches the standard value, the localized surface plasmon response of MoS_2_ reaches its maximum, and the charge transfer coupling effect between it and the graphene layer is fully activated, which strongly supplements the absorption in the mid-high frequency band (5.5–6.5 THz), ultimately making the entire ultra-wideband absorption platform flat.

#### 3.2.5. Discussion on Thermal Emission Coupling and System Stability

The proposed device requires heating VO_2_ through its insulator-metal transition point (approximately 341 K/68 °C) during coarse-tuning switching, while using ionic liquids as gate electrodes to finely control graphene and molybdenum disulfide. This dual-drive scheme may cause thermal emission coupling through two pathways. Firstly, local temperature increase to trigger the VO_2_ phase transition may affect the electrochemical stability of the ionic liquid gate electrolyte. However, the commonly used room-temperature ionic liquids for gate electrodes of two-dimensional materials (such as DEME-TFSI, EMIM-TFSI) still maintain good thermal and electrochemical stability within the range of 150–200 °C, which is much higher than the transition temperature of VO_2_. Therefore, under the operating conditions considered in this study, it is expected that no significant degradation of the electrolyte will occur. Secondly, although the Fermi level of graphene and the carrier concentration of molybdenum disulfide (MoS_2_) are mainly determined by the gate voltage, both exhibit weak secondary temperature dependence caused by thermal induced carrier scattering. This may lead to a drift of approximately 5% in the effective conductivity during the VO_2_ switching process.

In the strongly doped state at $E_{F}=0.8$ eV, the Fermi level of graphene is much higher than the room-temperature thermal excitation energy (300 K, $k_{B}T\approx 30.0\mathrm{meV}$). According to the Sommerfeld expansion, the absolute chemical potential thermal drift under the high Fermi level is satisfied $△\mu \approx -\left(\pi^{2}/6\right)\left(k_{B}T\right)/E_{F}$. When the environmental temperature rises from 298 K to 348 K, the calculated thermal drift amount of the internal chemical potential of graphene and MoS_2_ is less than 1.5 meV, with a relative drift rate of only 0.18%, and the Drude surface conductivity change caused in the terahertz frequency band is less than 0.5%, proving that the thermal crosstalk effect on the plane impedance of the two-dimensional heterojunction is very weak.

From a design perspective, the following methods can be employed to alleviate such crosstalk: (1) Utilize local micro-heaters or pulsed rather than continuous resistive/optical heating, limited to the VO_2_ layer, to minimize the diffusion of heat to the gate/electrolyte region. (2) Introduce a thin, low-thermal-conductivity buffer layer to thermally isolate the ionic liquid gate contact from the heating element. (3) Employ doping strategies such as W doping to reduce the oxygen consumption threshold temperature of VO_2_ to a level close to room temperature [30,31], thereby significantly reducing the required heating power and thermal gradient, and further suppressing thermal-electric crosstalk.

### 3.3. Incident Angle and Polarization Stability Evaluation

This section analyzes the variation in absorption with incident angle θ ($0^{\circ }-70^{\circ }$) under TE and TM polarizations.

**Figure 12:**
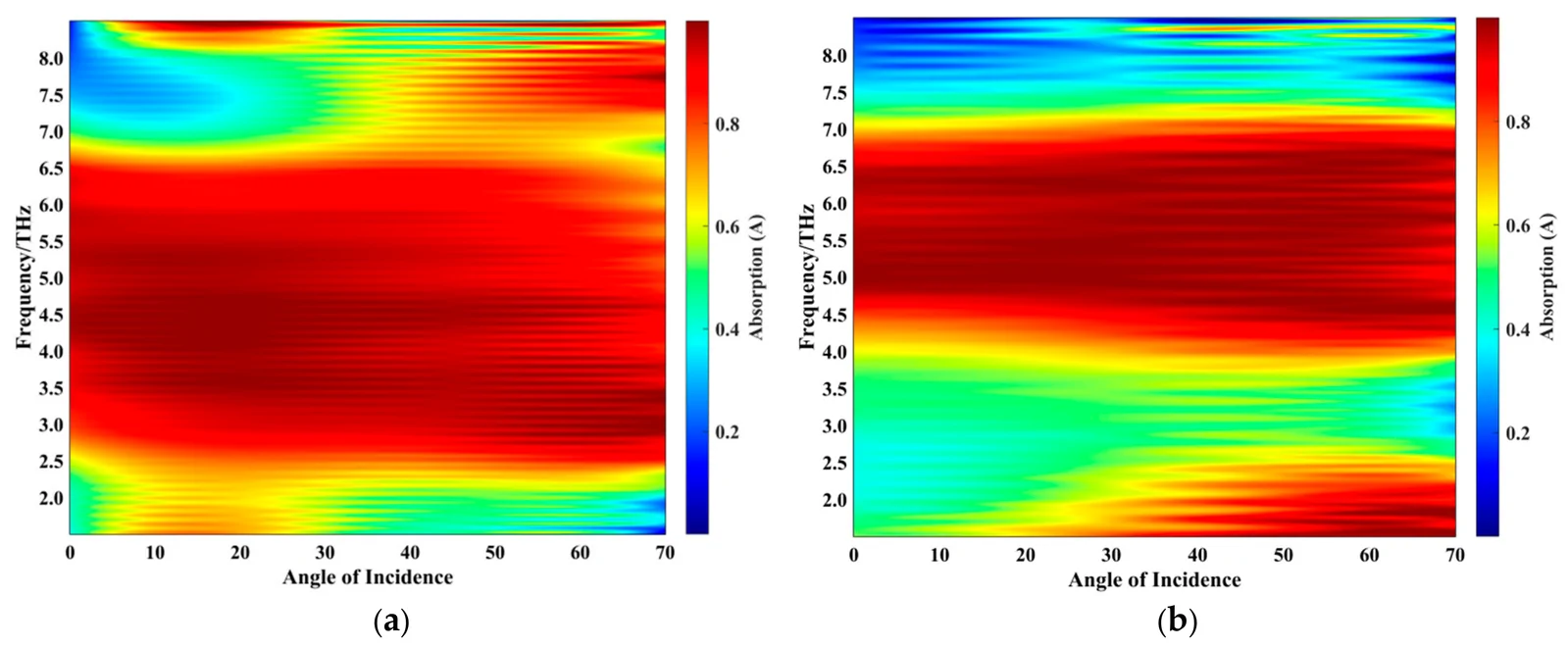
Stability of the incident angle on the absorber: (**a**) TE-polarized angular stability; (**b**) TM-polarized angular stability.

Figure 12a shows the evolution law of absorption with incident angle and frequency under TE polarization. The calculation data indicate that this structure has extremely strong angle robustness for TE polarization. When the incident angle increases from $0^{\circ }$ to $60^{\circ }$, the absorption within the 3.5–6.5 THz working frequency band remains above 91.7%, demonstrating extremely high efficiency stability. When the incident angle increases to $65^{\circ }$, the absorption band edge slightly contracts, but the peak absorption still remains above 87.55%. When the incident angle is $70^{\circ }$ the absorption in the high and medium frequency regions further decreases, remaining above 80%. However, the low and medium frequency regions can still maintain an absorption greater than 91.7%. This angle stability is related to the suppression of diffraction by subwavelength units and the weak dependence of local surface plasmons on the incident momentum.

Figure 12b shows the evolution law of absorption with incident angle and frequency under TM polarization. Compared with TE polarization, the working frequency band shifts to 4.7–6.6 THz under TM polarization, and the maximum absorption remains at 99.3%, and the working frequency band widens to 4.5–6.9 THz as the incident angle increases. This difference is mainly due to two factors. First, the incident magnetic field component under TM polarization is enhanced, coupling with the ring current in the asymmetric pattern on the top layer, exciting cross-layer magnetic resonance, and the magnetic resonance is more sensitive to the incident angle than the electric resonance. Second, the effective optical thickness of the VO_2_/PI longitudinal F-P cavity increases with the incident angle, causing the resonance frequency to shift to a lower frequency. The angle stability under TE polarization mainly comes from the suppression of parasitic diffraction by subwavelength units and the weak dependence of local surface plasmons on the incident momentum.

In summary, this absorber exhibits excellent stability for TE polarization over the full angle range, while for TM polarization, it shows angle-sensitive enhancement or degradation characteristics in the 4.7–6.6 THz frequency band.

### 3.4. Performance Comparison

To further evaluate the performance of the proposed absorber, Table 3 compares it with the recently reported terahertz absorbers based on VO_2_, graphene, molybdenum disulfide, and their mixed materials. The comparison focuses on the Operating frequency band, absorption rate, and angle stability. As shown in Table 2, the graphene/VO_2_ absorbers reported by Chen et al. [25] and Wen et al. [16] have a wider working frequency range and higher absorption rate, but their angle stability is not symmetrical in TE and TM polarizations. The graphene/MoS_2_ absorber reported by He et al. [20] has a relatively narrow working frequency range and limited angle stability at large incident angles. In contrast, the proposed graphene/MoS_2_/VO_2_ absorber has an absorption rate greater than 91.7% in the 3.5–6.5 THz range. In TE polarization, the absorption rate remains above 91.7% within the 0–60° incident angle range; in TM polarization, the maximum absorption rate reaches 99.3%, and the working frequency range shifts to 4.7–6.6 THz. These results indicate that, compared with the reported dual-material absorbers, the proposed absorber provides a better balance in terms of working frequency range, absorption rate, and angle stability.

## 4. Conclusions

This paper numerically demonstrates a THz broadband absorber integrating a graphene/MoS_2_ vertical heterojunction with a VO_2_ phase-change film, in which VO_2_ provides macroscopic coarse switching, graphene enables continuous electrical fine-tuning, and MoS_2_ offers low-loss field enhancement—to our knowledge, the first single-metasurface realization of this three-material synergistic hierarchy. In the VO_2_ insulating state, the device achieves >91.7% absorption over 3.5–6.5 THz (60.8% relative bandwidth, peak 98.75% at 4.43 THz), with <2% performance degradation under ±20% gap-size deviation, confirming strong process robustness. CMT decomposes the broadband response into three under-coupled modes (low-frequency anchoring, mid-frequency bridging, high-frequency precision anchoring; *R*^2^ = 0.9948), and unit-removal analysis confirms that the eight asymmetric units cooperate through near-field coupling and current-topology reconstruction rather than linear superposition. Heating VO_2_ into its metallic state suppresses absorption below ~49.5%, enabling broadband-absorption/reflection switching; graphene’s $E_{F}$ (0.2–0.8 eV) continuously broadens and blue-shifts the absorption band; and MoS_2_ carrier-concentration tuning further flattens the mid-to-high-frequency response. The absorber maintains >91.7% absorption for 0–60° incidence under TE polarization, with angle-dependent band-shifting behavior under TM polarization. These results provide a design reference for reconfigurable THz metasurfaces based on synergistic three-material integration; experimental validation via CVD growth, 2D-material transfer, and lithographic patterning, together with investigation of thermal–electrical crosstalk mitigation, is planned as part of future work.

## Figures and Tables

**Table 1:** The geometric parameters of each unit.

Unit	Graphene	MoS_2_
1	25 μm × 3 μm	32 μm × 5 μm
2	-	7 μm × 25 μm
3	9.5 μm × 2 μm	14.5 μm × 4 μm
4	3 μm × 14 μm	5 μm × 20 μm
5	5.2 μm × 3 μm	6 μm × 5 μm
6	3 μm × 3 μm	3 μm × 5 μm
7	14 μm × 3 μm	13 μm × 5 μm
8	24 μm × 25.5 μm	5 μm × 24.5 μm

**Table 2:** Physical parameters of each mode extracted by CMT fitting (all modes exhibit the under-coupled characteristic of $\gamma_{r}<\gamma_{a}$).

Mode	Resonance Frequency $\omega_{0}$ (THz)	Radiative Loss $\gamma_{r}$ (THz)	Absorptive Loss $\gamma_{a}$ (THz)	Quality Factor Q	Coupling Ratio $\gamma_{r}/\gamma_{a}$
1	3.178	0.4285	1.2312	1.91	0.348
2	5.200	0.5351	1.5490	2.50	0.345
3	6.594	0.1612	0.5411	9.39	0.298

**Table 3:** Comparison with reported dual-material terahertz absorbers.

Representative Work	Material System	Operating Frequency Band	Absorption	Angle Stability
Chen W. et al. (2022) [22]	Graphene + VO_2_ (six-layer)	2.85–10 THz	>95%	TE: ≤70°, TM: ≤50°
Wen Hao. et al. (2025) [21]	Graphene + VO_2_ (five-layer)	2.05–14.03 THz	>90%	TE: ≤50°, TM: ≤70°
He Jiayi. et al. (2026) [25]	Graphene + MoS_2_	2.1–3.9 THz	>90%	>85% at 40°
This Work	Graphene/MoS_2_/VO_2_	3.5–6.5 THz	>91.7%	TE: >91.7% within 0–60°; TM: 99.3% max, band shifts to 4.7–6.6 THz

## Data Availability

The original contributions presented in the study are included in the article. Further inquiries can be directed to the corresponding author.
